# Spatial gene expression profile of Wnt-signaling components in the murine enteric nervous system

**DOI:** 10.3389/fimmu.2024.1302488

**Published:** 2024-01-18

**Authors:** Melanie Scharr, Bernhard Hirt, Peter H. Neckel

**Affiliations:** Institute of Clinical Anatomy and Cell Analysis, University of Tübingen, Tübingen, Germany

**Keywords:** *in situ* hybridization, canonical Wnt signaling, non-canonical Wnt signaling, Dickkopf, R-Spondin, enteric nervous system, RNA sequencing, enteric gliosis

## Abstract

**Introduction:**

Wnt-signaling is a key regulator of stem cell homeostasis, extensively studied in the intestinal crypt and other metazoan tissues. Yet, there is hardly any data available on the presence of Wnt-signaling components in the adult enteric nervous system (ENS) *in vivo*.

**Methods:**

Therefore, we employed RNAscope HiPlex-assay, a novel and more sensitive *in situ* hybridization technology. By amplifying target specific signals, this technique enables the detection of low abundance, tightly regulated RNA content as is the case for Wnt-signaling components. Additionally, we compared our data to previously published physiological single cell RNA and RiboTag-based RNA sequencing analyses of enteric gliosis using data-mining approaches.

**Results:**

Our descriptive analysis shows that several components of the multidi-mensional regulatory network of the Wnt-signaling pathway are present in the murine ENS. The transport and secretion protein for Wnt-ligands Wntless as well as canonical (Wnt3a and Wnt2b) and non-canonical Wnt-ligands (Wnt5a, Wnt7a, Wnt8b and Wnt11) are detectable within submucosal and myenteric plexus. Further, corresponding Frizzled receptors (Fzd1, Fzd3, Fzd6, and Fzd7) and regulatory signaling mediators like R-Spondin/DKK ligands are present in the ENS of the small and large intestine. Further, data mining approaches revealed, that several Wnt-related molecules are expressed by enteric glial cell clusters and are dynamically regulated during the inflammatory manifestation of enteric gliosis.

**Discussion:**

Our results suggest, that canonical and non-canonical Wnt-signaling has a much broader impact on the mature ENS and its cellular homeostasis in health and inflammation, than previously anticipated.

## Introduction

The enteric nervous system (ENS) represents the intrinsic innervation of the gut and arises from migrating vagal neural crest cells originating from the neural tube. These precursor cells proliferate and differentiate into multiple subtypes of enteric neurons and glial cells. In the adult ENS of mammals, enteric neurons and glia are organized in two predominant networks of interconnected plexus (submucosal and myenteric), that are wired into microcircuits enabling gastrointestinal function independent of central nervous system control (1, 2). However, the ENS is still immature at birth and underlies considerable refinement including changes in its neurochemical coding as well as synaptic wiring to exert its proper function (3). Particularly the submucosal plexus develops late during embryogenesis and groups of neurons forming ganglia are not found before birth in the murine colon (4). In addition, a notable number of submucosal and myenteric progenitors undergo enteric neurogenesis within the first postnatal days in rodents (5, 6).

Unlike the early postnatal days, enteric neurogenesis in the adult ENS is controversially discussed and arguably a rare event in the submucosal and myenteric plexus *in vivo* (6–8). Although many *in vitro* studies (9–11) demonstrated a regenerative potential of postnatal-derived ENS-progenitor cells of various rodent species and human patients, only models of chemical denervation (12) and pathological settings like colitis (13) resulted in remodeling processes of the ENS *in vivo*. Still, the ever-changing environment of the gut, like constant physical stimuli by different motility patterns (14, 15), microbiome (16) or immunological interactions (17) suggest that cellular ENS-homeostasis might be continuously regulated by cell-cell-communication systems to maintain the steady state of the adult ENS.

In this context, enteric glial cells have gained increasing attention over the past years, as regulatory interaction partners in the reciprocal communication with enteric neurons (18), immune cells (19) and epithelial stem cells (20). Moreover, glial cell communication pathways involved in phenotypic plasticity induced by gastrointestinal disorders or systemic diseases are mostly unknown (21, 22).

During embryogenesis, Wnt-signaling induces and specifies neural crest formation and is involved in regulating enteric neural cell migration, guidance, and growth of enteric neuronal projections in the developing gut (23, 24). Further, Wnt-signaling is a key regulator of epithelial adult stem cell niches in different metazoan tissues including the gut epithelium (25), and the localization of Wnt-signaling components within the mucosa is well characterized (26). Intriguingly, a recent study by Baghdadi et al. highlighted the presence of enteric glial cells in the *Lamina propria mucosae*, which express several Wnt-signaling ligands to promote the self-renewal capacity of intestinal epithelial stem cells and thereby influence epithelial regeneration and barrier function in mice (20). Nevertheless, the presence of Wnt-signaling components in large parts of the adult ENS – especially in the submucosal and myenteric plexus *in vivo* has been largely neglected so far, and the functional evaluation is restricted to a handful of studies (27–29). Interestingly, the myenteric plexus is integrated into a network of myogenic cells, that are known to actively secrete Wnt-signaling molecules (30), conceivably shaping the cellular homeostasis and cytoarchitecture of the adult ganglionated plexus. Intriguingly, recently published transcriptome profiling data of the widespread used *in vitro* model system of purified ENS-progenitors, suggested, that mesenchymal cells indeed produced ligands belonging to the Wnt-signaling pathway (31). In particular, some of these ligands and corresponding receptors have roles in proliferation, differentiation, or maintenance of neural stem and progenitor cells (32, 33). Furthermore, regulatory Wnt-signaling co-activators like R-Spondins (34)- and Wnt-antagonists like Dickkopf (35)-ligands share heparin-binding properties, that enable interaction with the extracellular matrix component heparin sulfate proteoglycans. Since secretory heparin sulfate proteoglycans, such as collagen 18a and agrin, were shown to have an influence on proper ENS development in chicken (36), it is plausible that Wnt-components also interact with the extracellular matrix, assuming a profound influence on ENS-homeostasis.

To clarify the presence of Wnt-signaling components *in vivo*, we mapped the expression profile of these molecules in the adult murine small and large intestine. We take the advantage of a novel, sensitive *in situ* hybridization technology, commercially available as RNAScope HiPlex-assay. By amplify target-specific signals, this technique enables the detection of low abundance, tightly regulated RNA content as it is the case for the Wnt-signaling pathway. Further, to refine the cell type specific localization and contextualise our findings, we compared our RNAscope-based expression atlas to a recently published single cell RNA sequencing dataset of the juvenile (20 days of age) mouse ENS (37). Moreover, to assess the relevance of Wnt-signaling under pathophysiological conditions, we re-evaluated a RiboTag-based bulk RNA sequencing dataset of myenteric glial cells in acute inflammation (22) especially focusing on Wnt-signaling related targets. Thus, in this study, we extended the existing knowledge about the presence of Wnt-signaling components in the gastrointestinal tract by specifically focusing on the enteric nervous system. Our study suggests, that Wnt-signaling has a much broader impact on the adult ENS homeostasis in health and inflammation, than anticipated previously.

## Materials and methods

### Animals

Animals were handled and kept in accordance with the guidelines, regulating the handling of animals for scientific purposes (TierVersV, Notification number AT 01/19 M), which conform to international guidelines. C57BL/6J were housed in standard cages with standard pathogen-free breeding and a standard 12-hour light/dark cycle at 22 ± 2°C and 60 ± 5% humidity. Germ-free food and water were available ad libitum. For *in situ* hybridization experiments, small and large intestine samples from male C57BL/6J mice (postnatal day 60) were used.

### *In situ* hybridization: fixed frozen tissue sample preparation, pre-treatment, RNAscope^®^ HiPlex Assay and detection

We applied the RNAscope^®^ HiPlex Assay and HiPlex Assay v2 (ACB biotechne, Wiesbaden-Nordenstadt Germany) according to the manufacturer’s description to detect expression of relevant Wnt-ligands and receptor mRNAs in small and large intestine samples.

### Sample preparation

The intestine bundle was removed from the abdomen, transferred to and rapidly dissected in 4% (w/v) phosphate buffered p-formaldehyde (Merck KGaA, Darmstadt, Germany). Adherent mesenteria were dissected to unfold intestine. Afterwards, small, and large intestine were separated and for better handling cut into 2 cm long samples.

### Fixed frozen tissue sample preparation and pre-treatment

Before embedding, prepared tissue samples were fixed with 4% (w/v) phosphate buffered p-formaldehyde (Merck KGaA, Darmstadt, Germany) at 4°C for one hour and rinsed 3 times with RNAse-free phosphate-buffered saline (PBS). Afterwards, fixed samples were stored overnight at 4°C in 30% (w/v) sucrose solution (AppliChem, Darmstadt, Germany). Next, samples were frozen in isopentane-nitrogen cooled TissueTek^®^ (Sakura, Staufen, Germany) in such a way that, three transversal small and large intestine samples were on one section. Blocks were stored at -80°C until further processing. Prepared cryosections of 12 µm thickness were washed with PBS and dried for 30 minutes at 60°C, following post-fixation with 4% (w/v) phosphate buffered p-formaldehyde for 15 minutes at 4°C. Then, sections were dehydrated in an ascending alcohol series: each step for 5 minutes in 50% (v/v), 70% (v/v) and twice in 100% (v/v) EtOH at room temperature. Target retrieval was performed in mild-boiling (98 – 102°C) 1x RNAscope^®^ Target Retrieval Reagent solution for 5 minutes, followed by one washing step in distilled water and one in 100% (v/v) EtOH. Sections were dried completely at 60°C for 5 minutes, before the hydrophobic barrier was drawn. One the next day, RNAscope^®^ Protease III treatment was applied for 5 minutes at 40°C. Before continuing with the RNAscope^®^ HiPlex Assay, sections were washed once with distilled water.

### RNAscope^®^ HiPlex assay

Prior probe hybridization, pre-warmed RNAscope^®^ HiPlex 50x probe stocks were diluted with RNAscope^®^ HiPlex Diluent according to the manufacturer’s description and cooled to room temperature prior to use. Probe hybridization was carried out at 40°C for 2 hours. Afterwards, sections were washed twice for two minutes at room temperature with 1x RNAscope^®^ Wash Buffer. Next, three amplification rounds were carried out, while sections were treated successively first with the RNAscope^®^ HiPlex Amp1, second the RNAscope^®^ HiPlex Amp2 and third with the RNAscope^®^ HiPlex Amp3 for 30 minutes at 40°C. After each amplification round, sections were washed twice with 1x RNAscope^®^ Wash Buffer for 2 minutes at room temperature.

For detection, sections were treated 15 minutes with the corresponding RNAscope^®^ HiPlex Fluor Solution at 40°C, washed twice with 1x RNAscope^®^ Wash Buffer for 2 minutes at room temperature, counterstained with RNAscope^®^ DAPI for 1 minute and mounted with ProLong Gold Antifade Mountant (Thermo Fisher Scientific, MA, USA). For each experiment a positive and a negative control was performed to evaluate signal strength as well as background staining. Target probe signal was evaluated according to the manufacturer’s description. Commercially designed, generated, and evaluated target probes and their corresponding probe channel used in this study are listed in **Tables 1**, **2**.

**Table 1:** HiPlex Target probes used in this study.

Target probe	Channel
Mus musculus dickkopf homolog 1 (Xenopus laevis) (Dkk1)	Atto647N
Mus musculus dickkopf homolog 2 (Xenopus laevis) (Dkk2)	AF488
Mus musculus dickkopf homolog 3 (Xenopus laevis) (Dkk3)	Atto550
Mus musculus dickkopf homolog 4 (Xenopus laevis) (Dkk4)	Atto647N
Mus musculus frizzled homolog 1 (Drosophila) (Fzd1)	Atto647N
Mus musculus frizzled homolog 2 (Drosophila) (Fzd2)	AF488
Mus musculus frizzled homolog 3 (Drosophila) (Fzd3)	Atto647N
Mus musculus frizzled homolog 4 (Drosophila) (Fzd4)	AF488
Mus musculus frizzled homolog 5 (Drosophila) (Fzd5)	Atto550
Mus musculus frizzled homolog 6 (Drosophila) (Fzd6)	Atto647N
Mus musculus frizzled homolog 7 (Drosophila) (Fzd7)	Atto550
Mus musculus frizzled homolog 8 (Drosophila) (Fzd8)	AF488
Mus musculus frizzled homolog 9 (Drosophila) (Fzd9)	Atto550
Mus musculus frizzled homolog 10 (Drosophila) (Fzd10)	Atto647N
Mus musculus Glycerinaldehyd-3-phosphat-Dehydrogenase (GAPDH)	Atto647N
Mus musculus Hypoxanthine phosphoribosyltransferase 1 (HPRT)	AF488
Mus musculus kringle containing transmembrane protein 1 (Kremen1)	Atto647N
Mus musculus kringle containing transmembrane protein 2 (Kremen2)	Atto550
Mus musculus leucine rich repeat containing G protein coupled receptor 4 (Lgr4)	Atto550
Mus musculus leucine rich repeat containing G protein coupled receptor 5 (Lgr5)	AF488
Mus musculus leucine-rich repeat-containing G protein-coupled receptor 6 (Lgr6)	AF488
Mus musculus low density lipoprotein receptor-related protein 5 (Lrp5)	AF488
Mus musculus low density lipoprotein receptor-related protein 6 (Lrp6)	Atto550
Mus musculus R-spondin 1 homolog (Xenopus laevis) (Rspo1)	Atto647N
Mus musculus R-spondin 2 homolog (Xenopus laevis) (Rspo2)	AF488
Mus musculus R-spondin 3 homolog (Xenopus laevis) (Rspo3)	Atto550
Mus musculus R-spondin family, member 4, transcript variant 1 (Rspo4)	Atto550
Mus musculus Polyubiquitin-C (UBC)	Atto647N

**Table 2:** HiPlex Target probes used in this study.

Target probe	Channel
Mus musculus wntless homolog (Drosophila), transcript variant 1 (Wls)	AF488
Mus musculus wingless-related MMTV integration site 1 (Wnt1)	AF488
Mus musculus wingless-related MMTV integration site 2 (Wnt2)	Atto647N
Mus musculus wingless-related MMTV integration site 2b (Wnt2b)	Atto550
Mus musculus wingless-related MMTV integration site 3 (Wnt3)	AF488
Mus musculus wingless-related MMTV integration site 3a (Wnt3a)	Atto550
Mus musculus wingless-related MMTV integration site 4 (Wnt4)	AF488
Mus musculus wingless-related MMTV integration site 5a, transcript variant 1, (Wnt5a)	Atto647N
Mus musculus wingless-related MMTV integration site 5b, transcript variant 2 (Wnt5b)	AF488
Mus musculus wingless-related MMTV integration site 6 (Wnt6)	Atto550
Mus musculus wingless-related MMTV integration site 7a (Wnt7a)	Atto647N
Mus musculus wingless-related MMTV integration site 7b, transcript variant 1 Wnt7b)	AF488
Mus musculus wingless-related MMTV integration site 8a (Wnt8a)	Atto550
Mus musculus wingless-related MMTV integration site 8b (Wnt8b)	Atto647N
Mus musculus wingless-related MMTV integration site 9a (Wnt9a)	Atto550
Mus musculus wingless-related MMTV integration site 9b (Wnt9b)	Atto647N
Mus musculus wingless-related MMTV integration site 10a (Wnt10a)	AF488
Mus musculus wingless-related MMTV integration site 10b (Wnt10b)	Atto647N
Mus musculus wingless-related MMTV integration site 11 (Wnt11)	AF488
Mus musculus wingless-related MMTV integration site 16 (Wnt16)	Atto550

### Microscopy

Images were acquired using a Zeiss Axio Imager.Z1 fluorescence microscope with Apotome module with 358, 488, 543, 647 nm for excitation and appropriate filter sets. Images were acquired using ZEN software. For *in situ* hybridization exposure time for DAPI was 150 ms and for the RNAscope HiPlex Fluoro solutions 5000 ms with the 63-objective (Plan-Apochromat 63x/1.40 Oil DIC M27). RNAscope demonstrated a clear punctate staining, whereby each RNA dot derives from a single mRNA molecule.

Hybridization probes targeting housekeeping genes, including GAPDH (Glycerin-aldehyd-3-phosphat-Dehydrogenase), HPRT (Hypoxanthine phosphoribosyl trans-ferase 1) and UBC (Polyubiquitin-C), were used as positive controls for the hybridization procedure in each independent preparation. Samples treated with dapB probes were used as negative controls to assess background signals (**Supplementary Figures 1A, B** for negative control).

We applied a semiquantitative evaluation, whereby less than five dots correspond to no expression. Low expression is marked by single mRNA-transcripts with less signal intensity, that are clearly recognizable from the tissue background. mRNA transcripts displaying a strong signal intensity, still with clearly distinguishable single dots were defined as medium expression. High expression is characterized by a high density of no separable single dots. The distal jejunum and ileum of the small intestine as well as the proximal half of the large intestine was included in our study, whereby we evaluated the following regions: epithelium (w/o crypts), epithelium (crypts), *Tunica muscularis*, as well as submucosal and myenteric ganglia. In the small intestine the category *epithelium (w/o crypts)* covers the area from the tip of the villus surface down to the crypt-villus junction. In the large intestine the *epithelium (w/o crypts)* category is defined by the complete epithelial surface excluding the crypts. The category *epithelium (crypts)* includes the entire crypts from the crypt-villus junction down to the crypt bottom in both small and large intestine. If applicable more precise localization was described in the text.

## Results

In this study we employed the RNAscope HiPlex-assay, a novel and more sensitive *in situ* hybridization technology to investigate components of the Wnt signaling network particularly focusing on the ENS of adult P60 mice. This method uses a special amplification procedure that enables the detection of single mRNA transcripts, making it especially useful for the evaluation of low abundance transcripts of tightly regulated signaling cascades. Further, we compared our data to previously published physiological single cell RNA (37) and RiboTag-based RNA (22) sequencing analysis of acute inflammation using data-mining approaches.

### *In vivo* expression of Wnt-ligands in mouse intestine

**Figure 1:**
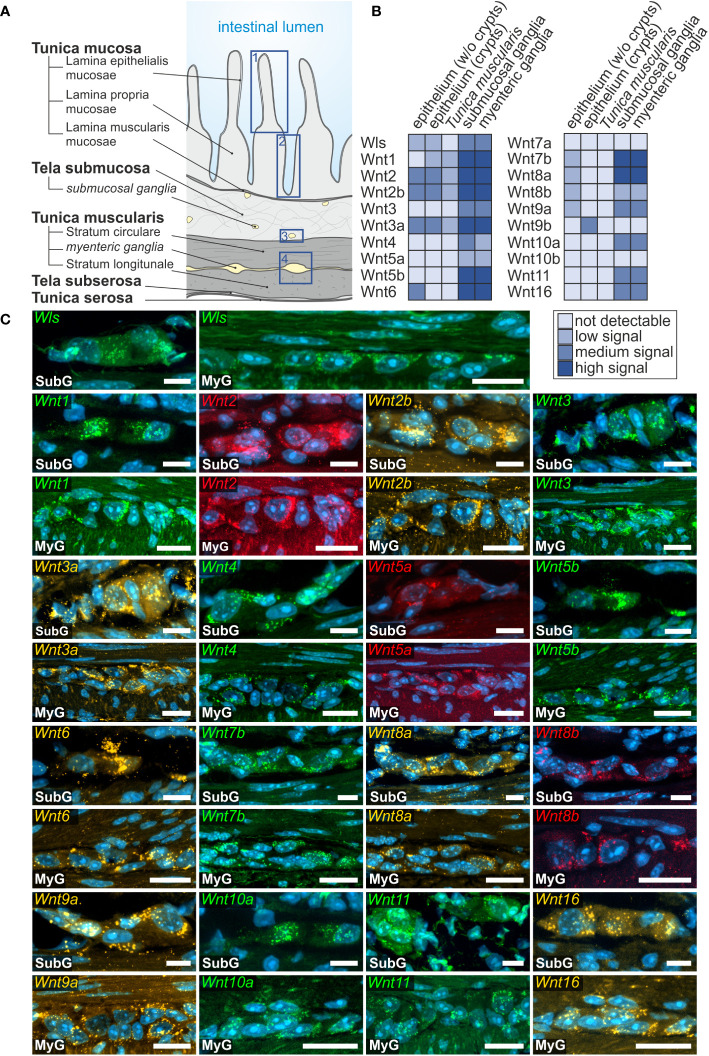
Wnt-ligands expressed in the murine small intestine. **(A)**: Schematic drawing of the gut wall with the histological landmarks. Numbered rectangles (dark blue) indicate the regions examined: 1: epithelium (without crypts); 2: epithelium (only crypt region); 3: submucosal ganglia; 4: myenteric ganglia with surrounding musculature. **(B, C)**: *In situ* hybridization experiments showed *Wls-* as well as *Wnt1-, Wnt2-, Wnt2b-, Wnt3-, Wnt3a-, Wnt4-, Wnt5a-, Wnt5b-, Wnt6-, Wnt7b-, Wnt8a-, Wnt8b-, Wnt9a-, Wnt10a-, Wnt11-* and *Wnt16*-mRNAs detection (colors as indicated) and the nuclear marker DAPI (blue) within submucosal (SubG) and myenteric plexus (MyG) of murine small intestine with different expression levels. Scale bars: 40 µm.

**Figure 2:**
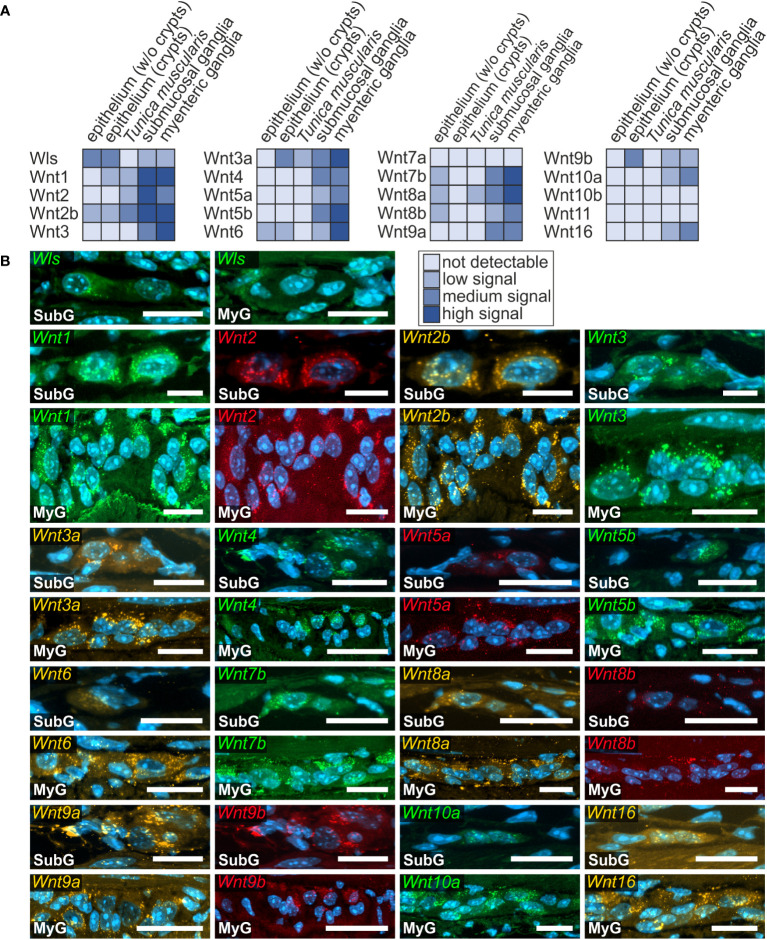
Presence of Wnt-ligand-transcripts in the murine large intestine. **(A, B)**: *Wls-* as well as *Wnt1-, Wnt2-, Wnt2b-, Wnt3-, Wnt3a-, Wnt4-, Wnt5a-, Wnt5b-, Wnt6-, Wnt7b-, Wnt8a-, Wnt8b-, Wnt9a-, Wnt9b-, Wnt10a-* and *Wnt16*-transcripts (colors as indicated) were found within submucosal (SubG) and myenteric plexus (MyG) of murine large intestine with different expression levels. Cell nuclei are stained for the nuclear marker DAPI (blue). Scale bars: SubG 20 µm and MyG 40 µm.

Overall, we found a broad expression of Wnt-ligand mRNAs in enteric ganglia, with a majority of probes generating clearly detectable signals in the small (16 out of 19) and large intestine (13 out of 19). Additionally, we detected the expression of the multi-pass transmembrane protein Wntless/Evi, which is essential for intracellular Wnt-shuttling and Wnt-secretion of lipidated Wnt-ligands (38, 39) in the submucosal and myenteric plexus of the small intestine (**Figure 1**). In contrast to the small intestine, Wntless/Evi expression was less abundant and restricted to some cells within enteric ganglia of the colon (**Figure 2**). In the small intestine, we found the expression of *Wnt1-, Wnt2-, Wnt2b-, Wnt3-, Wnt3a-*, *Wnt4-, Wnt5a-*, *Wnt5b-*, *Wnt6-*, *Wnt7b-*, *Wnt8a-*, *Wnt8b-*, *Wnt9a-, Wnt10a*-, *Wnt10b-, Wnt11-*, and *Wnt16*-transcripts in the submucosal (**Figures 1B, C** - SubG) and myenteric (**Figures 1B, C** – MyG) plexus. The signal intensity was comparable for all detectable transcript, excluding *Wnt4-*, *Wnt5a-*, and *Wnt8b-*mRNAs, which exhibited considerably lower, yet clearly distinguishable, signal levels (**Figure 1B**). Interestingly, some Wnt-ligands, such as *Wnt1*, *Wnt2*, and *Wnt2b*, showed a preferential expression in myenteric neuronal somata as compared to glial cell bodies and surrounding ganglionic neuropil (**Supplementary Table 1**). In contrast, most other transcripts did not exhibit a clear localization to a specific cell type (**Figure 1C**). Moreover, *Wnt7a-*, *Wnt9b-*, and *Wnt10b-*transcripts were not detectable in the ganglia neither in the submucosal nor in the myenteric plexus (**Supplementary Figure 1C**, **Supplementary Table 1**).

To put our results into a wider context with previously published RNA expression profiles, we compared our RNAscope-based expression atlas (**Supplementary Table 1**) of the small intestine to a preexisting single cell RNA sequencing dataset of the juvenile murine myenteric plexus (38). Interestingly, there we found that *Wnt2*-, *Wnt2b, Wnt3* and *Wnt16* transcripts were only detectable in the myenteric neuronal cell cluster, whereas *Wnt5b*, *Wnt7b* and *Wnt10a* mRNAs were observed in the glial cell cluster. Further, detectable mRNA expression of *Wls*, *Wnt1*, *Wnt4*, *Wnt5a*, *Wnt6*, *Wnt9a*, as well as *Wnt11* was found within both, neuronal and glial cell clusters. *Wnt3a-*, *Wnt7a-*, *Wnt8a-*, *Wnt8b-*, *Wnt9b-* and *Wnt10b*-transcripts were not found in the neuronal nor glial clusters (**Supplementary Table 2**).

In the large intestine, we found the expression of *Wnt1-, Wnt2-, Wnt2b-, Wnt3-, Wnt3a-*, *Wnt4-, Wnt5a-*, *Wnt5b-*, *Wnt6-*, *Wnt7b-*, *Wnt8a-*, *Wnt8b-*, *Wnt9a-, Wnt9b-*, *Wnt10a*-, and *Wnt16*-transcripts throughout myenteric ganglia, however, with variable intensities (**Figure 2A**). While Wnt5a, Wnt8b, and Wnt9b were low, yet clearly abundant, the signal intensity of the remaining detectable Wnt-ligands was considerably stronger (**Figure 2B**). In addition, *Wnt1-, Wnt2-, Wnt2b-, Wnt3-, Wnt3a-*, *Wnt4-, Wnt5a-*, *Wnt5b-*, *Wnt6-*, *Wnt7b-*, *Wnt8a-*, *Wnt8b-*, *Wnt9a-, Wnt9b-*, *Wnt10a*-, and *Wnt16*-transcripts were also found in submucosal plexus, though we found *Wnt5b-* and *Wnt6* mRNAs only found single submucosal neurons (**Figure 2B**). Moreover, *Wnt2* expression was noticeably more intense in the submucosal than in the myenteric ganglia. In contrast to the small intestine, *Wnt7a-*, *Wnt10b*-, and *Wnt11-*mRNAs were not detectable in the ganglia of the large intestine (**Supplementary Figure 1C**, **Supplementary Table 1**). Yet, like the findings in the small intestine, Wnt1, Wnt2, and Wnt2b exhibited a neuronal localization, both in submucosal and myenteric ganglia. Additionally, the expression of Wnt4 appeared to be restricted to a subset of neuronal somata in the myenteric plexus (**Figure 2B**).

Several Wnts-ligands were also expressed in other compartments of the intestines, including the epithelium, *Lamina propria mucosae* as well as in the smooth musculature of the *Tunica muscularis* of small (**Supplementary Figure 2**) and large intestine (**Supplementary Figure 3**). Since there is extensive data on the expression of Wnt signaling components in the intestinal epithelium and crypts, we used these compartments as internal positive controls, further validating our findings in the ENS. In the small intestine, transcripts of *Wnt2* and the closely related homolog *Wnt2b* were found in the epithelium (**Supplementary Figures 2A-i-ii, B-i-ii**, arrowheads), and cells of the *Lamina propria mucosae* (**Supplementary Figures 2A-i-ii, B-i-ii**, arrow), as well as within the *Tunica muscularis* (**Supplementary Figures 2A-iii, B-iii**, arrowheads).

In contrast, in the large intestine *Wnt2* and *Wnt2b* were less abundant. *Wnt2* was hardly expressed in the majority of epithelial cells (**Supplementary Figures 3A-i**, arrowheads) and exhibited a low expression profile throughout the *Lamina propria mucosae* (**Supplementary Figures 3A-ii**, arrowheads) and *Tunica muscularis* (**Supplementary Figures 3A-iii**, arrowheads). *Wnt2b* showed slightly higher expression levels, both in the epithelium as well as the *Tunica muscularis* (**Supplementary Figures 3B-i–B-iii**, arrowhead).

Furthermore, *Wnt3a*-transcripts were strongly expressed throughout the *Tunica mucosa* of the small intestine (**Supplementary Figures 2C-i-ii**, arrowhead), but was restricted to the crypt bottom in the large intestine (**Supplementary Figure 3C**). *Wnt6*-expression was found in the small intestine throughout the villus but was undetectable in the crypt bottom (**Supplementary Figure 2D**). In the large intestine, however, Wnt6 was expressed in the epithelium (**Supplementary Figures 3D-i**, arrowhead), including the crypt bottom (**Supplementary Figures 3D-ii**, arrowhead). *Wnt7b*-, *Wnt8a*-, and *Wnt8b*-transcripts were detectable with low expression rates in a subset of superficial epithelial cells (**Supplementary Figures 3E, F-i, G**, arrowhead). However, we did not detect any expression of these transcripts in the epithelial cells of the crypts, both in small and large intestine. Interestingly, we found a population of cells in the *Lamina propria mucosae* with high expression profiles of *Wnt7b* und *Wnt8a*, that lacked a detectable signal for *Wnt8b*, both in the small intestine (**Supplementary Figures 2E, F** arrow) and colon (**Supplementary Figures 3E, F-i** arrows). Moreover, *Wnt8a* was also expressed in the *Tunica muscularis* in the large intestine (**Supplementary Figures 3F-ii**, arrowhead). Finally, *Wnt9a* was located throughout the epithelium of the small intestine (**Supplementary Figure 2H**, arrowhead), whereas *Wnt9b*-transcripts were found at the crypt bottom of small and large intestine only (**Supplementary Figures 2I**, **3H**, arrowhead).

Wntless, the transport and secretion protein for Wnt-ligands, was found within the *Lamina propria mucosae* and occasionally in epithelial cells of small and large intestine (**Supplementary Figures 2J**, **3I**). Beside their expression within the enteric nervous system, neither *Wnt1-, Wnt3-, Wnt4-, Wnt5a-* and *Wnt5b-* and *Wnt10a-*transcripts, nor *Wnt1-, Wnt3-, Wnt5a-* and *Wnt5b-, Wnt10a-* and *Wnt16*-mRNAs were detectable within small and large intestine.

### Wnt-receptor expression in murine small and large intestine

**Figure 3:**
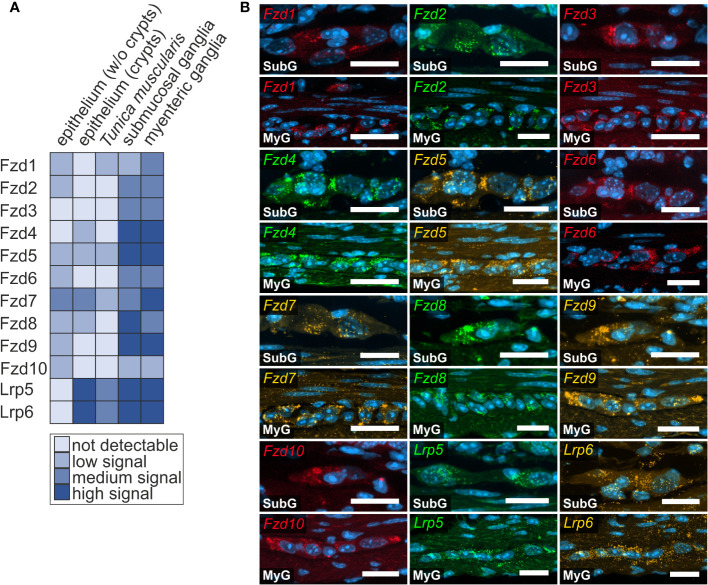
Frizzled-mRNAs in murine small intestine. **(A, B)**: *In situ* hybridization experiments showed *Fzd1-, Fzd2-, Fzd3-, Fzd4-, Fzd5-, Fzd6-, Fzd7-, Fzd8-, Fzd9-, Fzd10-, Lrp5- and Lrp6-*transcripts detection (colors as indicated) and the nuclear marker DAPI (blue) within submucosal (SubG) and myenteric plexus (MyG) of murine small intestine with different expression levels. Scale bars: 40 µm.

**Figure 4:**
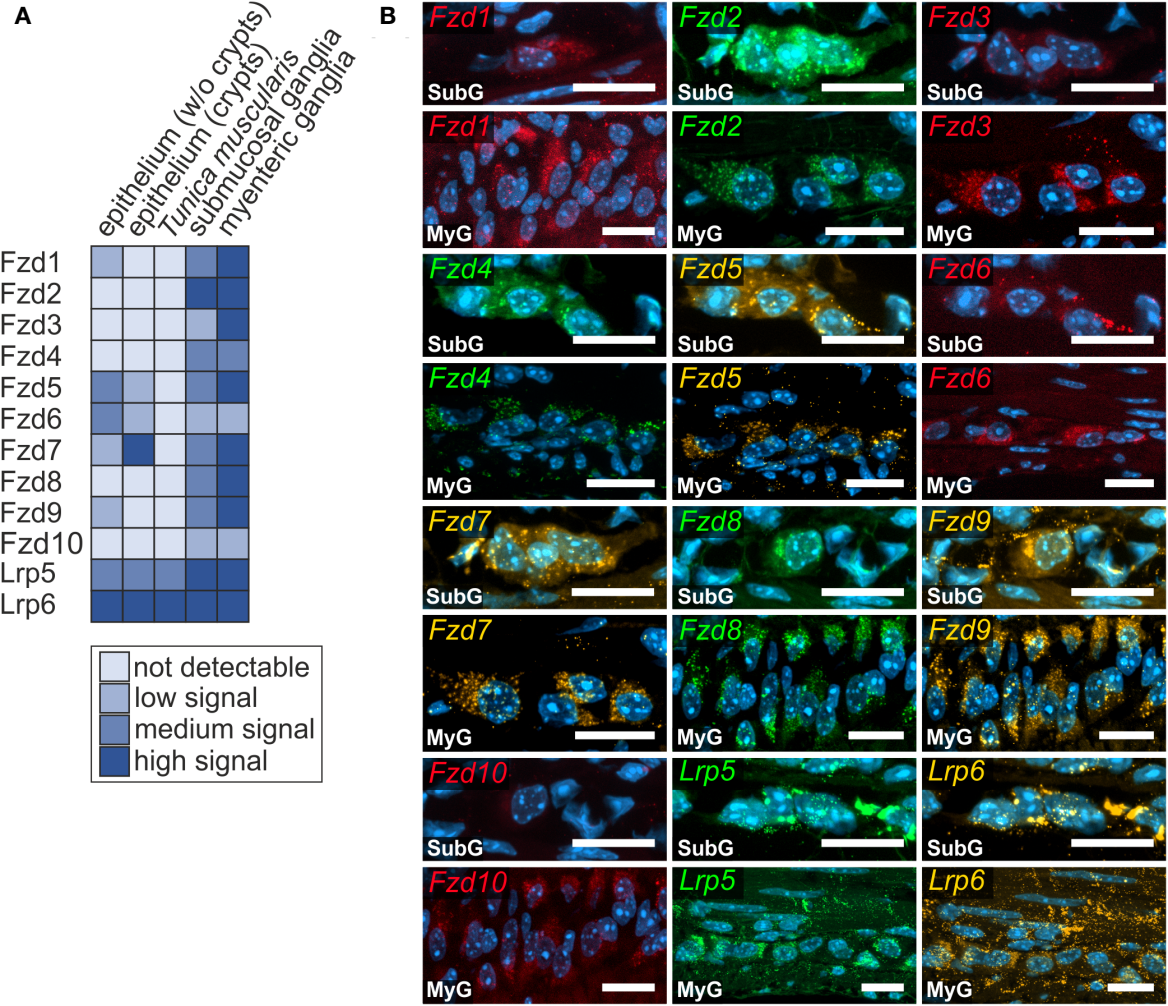
Frizzled-mRNAs are detectable in murine large intestine. **(A, B)**: *Fzd1-, Fzd2-, Fzd3-, Fzd4-, Fzd5-, Fzd6-, Fzd7-, Fzd8-, Fzd9-, Fzd10-, Lrp5- and Lrp6-*transcripts were detected (colors as indicated) within submucosal (SubG) and myenteric plexus (MyG) of murine large intestine with different intensity levels. Cell nuclei are stained with the nuclear marker DAPI (blue). Scale bars: SubG 20 µm and MyG 40 µm.

Next, we examined the expression of Frizzled receptors 1-10 as well as Lrp-receptors 5 and 6 to identify Wnt-responsive cells within the enteric nervous system. As shown in **Figure 3**, *Fzd*- and *Lrp*-transcripts were found within submucosal (**Figures 3A, B** - SubG) and myenteric ganglia (**Figures 3A, B** – MyG) of the small intestine and were also widely distributed within submucosal (**Figures 4A, B** – SubG) and myenteric (**Figures 4A, B** – MyG) ganglia of the large intestine. In the small intestine, the expression intensity was comparable for all transcripts, excluding *Fzd1-*, and Fzd10*-*mRNAs, which exhibited considerably lower, but clearly distinguishable signal levels in myenteric, but particularly in submucosal ganglia. Interestingly, some Fzd-receptors, such as *Fzd2*, *Fzd3*, *Fzd6*, and *Fzd7* showed a clearly recognizable expression in myenteric neuronal somata. In contrast, most other transcripts did not exhibit a clear localization to a specific cell type (**Figure 3**, **Supplementary Table 1**). Compared to the small intestine, expression signals of Fzd-transcripts were considerably stronger in the large intestine. Additionally, like the findings in the small intestine, *Fzd2*, *Fzd3*, *Fzd6*, and *Fzd7* displayed a neuronal localization, both in submucosal (**Figure 4B** – SubG) and myenteric (**Figure 4B** – MyG) ganglia. The transcripts of the co-receptors Lrp5 and Lrp6 exhibited highly intense signal levels throughout the myenteric and submucosal ganglia of the small intestine (**Figure 3**) and the colon (**Figure 4**). Again, comparing our results to scRNA-seq atlas of the juvenile murine myenteric plexus (38), we found a considerable mRNA expression of the *Fzd1-10 and Lrp5* and *Lrp6* transcripts within neuronal and glial cell cluster (**Supplementary Table 2**), which is partly in line with our own findings.

We further looked for the expression of *Fzd-* and *Lrp-*transcripts in other layers of the gut wall. We found a low hybridization signal for *Fzd1* throughout the epithelium of small (**Supplementary Figure 4A**) and large intestine (**Supplementary Figure 5A**). Interestingly, we detected a medium to high expression of *Fzd2*-, *Fzd5*-, *Fzd7*-, *Fzd8*-, and *Fzd9*-transcripts in a subset of cells in the *Lamina propria mucosae* of the small intestine (**Supplementary Figures 4B, D-i, F, G, H**, arrow), whereas in the large intestine only *Fzd9* was detectable at a comparable location (**Supplementary Figure 5E**, arrow). Occasionally, we also found *Fzd10-*transcritps localized within cells of the *Lamina propria mucosae* (**Supplementary Figure 4I**, arrow) as well as in a subset of epithelial cells of the small intestine (**Supplementary Figure 4I**, arrowhead). In the epithelium, there was a clearly visible concentration of cells expressing Fzd4, Fzd5, Fzd6, and Fzd7 in the small intestinal crypt bottom. While Fzd7 exhibited a partly similar expression at the crypt bottom of the colon, Fzd5 and Fzd6-expressing cells were absent throughout the crypt and rather located at the luminal surface (**Supplementary Figures 5B, C**, arrowhead). Except for the ENS, Fzd3 was not detectable in other parts of the gut wall both in the large and small intestine.

In contrast to Fzd-receptors, Lrp-receptors showed a stronger expression signal throughout all intestinal layers examined. Intriguingly, *Lrp5* was found within epithelial cells of the crypt bottom in the small intestine (**Supplementary Figures 4J-i**), whereas in the large intestine it was located in the cells of the *Lamina propria mucosae* surrounding the crypts (**Supplementary Figures 5F-i-ii**). *Lrp6*, on the other hand, exhibited an intense expression both, in the epithelial cells of the crypt bottom as well as the *Lamina propria* cells throughout the small intestine and colon (**Supplementary Figures 4K-i-ii**, **5G-i-iii**).

### Expression of the secreted Wnt-antagonist Dickkopf in murine small and large intestine

**Figure 5:**
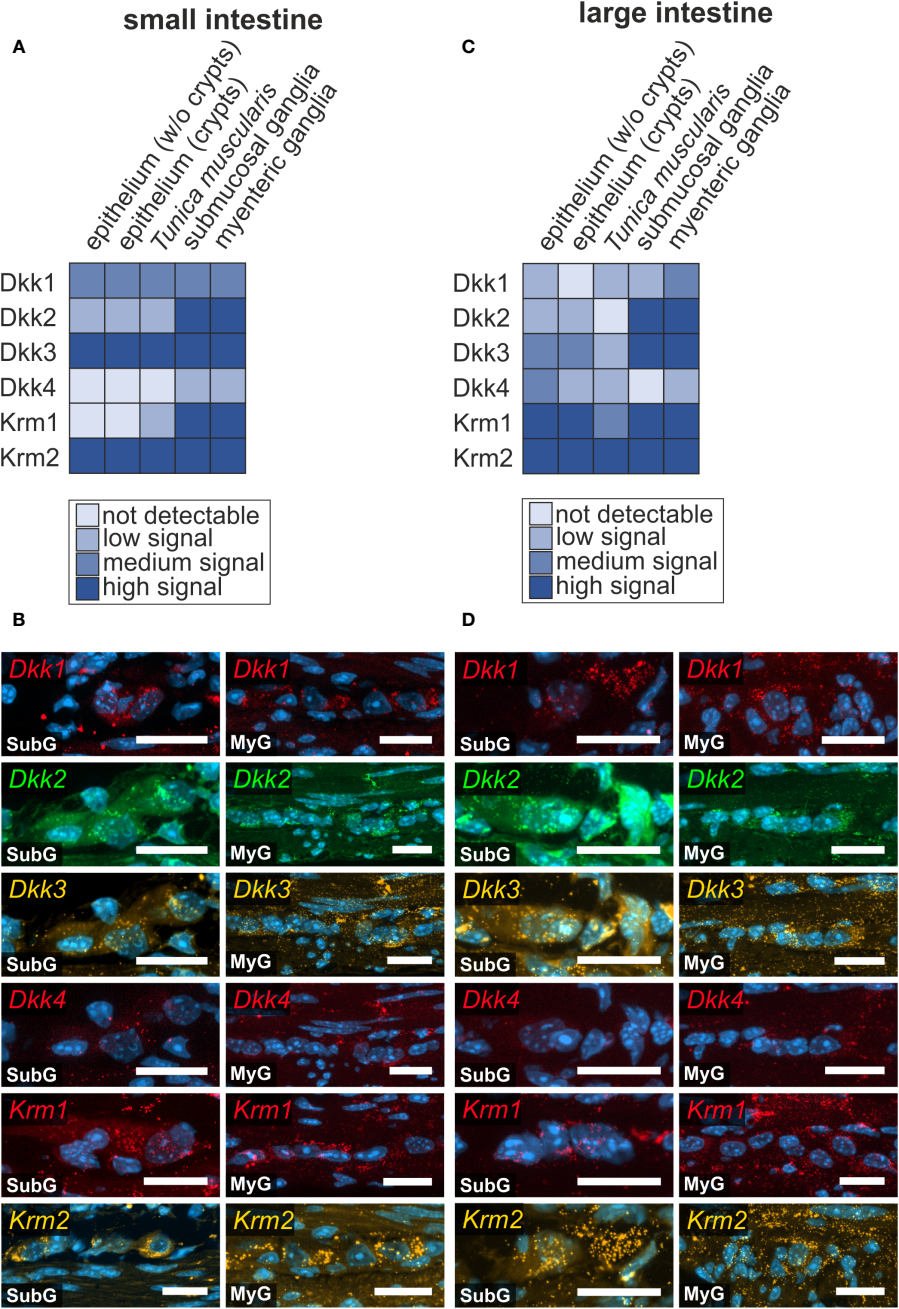
Prominent Wnt-signaling antagonists are expressed in the murine intestine. **(A, B)**: *Dkk1, Dkk2, Dkk3, Dkk4, Krm1* and *Krm2* mRNAs were detected with *in situ* hybridization experiments (colors as indicated) and the nuclear marker DAPI (blue) within submucosal (SubG) and myenteric plexus (MyG) of murine small intestine with different signal intensities. Scale bars: 40 µm. **(C, D)**: *Dkk1, Dkk2, Dkk3, Dkk4, Krm1* and *Krm2* transcripts (colors as indicated) and the nuclear marker DAPI (blue) were also detectable within myenteric plexus of murine large intestine Scale bars: SubG 20 µm and MyG 40 µm.

As a classical functional counterpart of activating Wnt-signals, we screened for classical secreted Wnt-antagonists, namely Dickkopf-ligands 1-4 *(Dkk1, Dkk2, Dkk3, Dkk4)*, and the relevant receptors Kremen 1-2 (*Krm1*, *Krm2)* and Low-density lipoprotein receptor-related protein 5 and 6 *(Lrp5* and *Lrp6)*, (see also **Figures 3**, **4**)*. Dkk*-ligands and corresponding receptors were detectable within the submucosal and myenteric plexus of the small (**Figures 5A, B**) and large intestine (**Figures 5C, D**). In the small intestine, Dkk-ligands showed variable expression levels within the myenteric plexus, in that *Dkk2-* and *Dkk3-*transcripts exhibited a more abundant and stronger expression than *Dkk1-* and *Dkk4-*mRNAs. Moreover, we observed that *Dkk4*-signals were weaker than *Dkk1-*transcripts (**Figure 5B**), and nearly absent in submucosal ganglia. This expression pattern was comparable in the small and large intestine (**Figure 5D**). Interestingly, *Krm1-* and *Krm2*-transcripts showed a higher expression intensity in myenteric ganglia of the large intestine than in small intestinal ganglia. Noteworthy, Dkk-ligands and their receptors did not exhibit a detectable preference to a specific ganglionic cell type (**Figures 5B, D** as well as **Supplementary Table 1**).

Similar results were obtained from single cell RNA sequencing. In detail, mRNA expression of *Dkk1-, Dkk2-, Dkk3-, Krm1*, as well as *Krm2* was found within the neuronal and glial cell clusters. In contrast to our dataset, *Dkk4* transcripts were not found in the neuronal and glial cluster (**Supplementary Table 2**).

*Dkk*- and *Krm-*expression was also observed within other layers of the intestinal wall of small intestine (**Supplementary Figure 6**) and large intestine (**Supplementary Figure 7**). In the small intestine, *Dkk1-* and *Dkk3-*transcripts were expressed throughout the villus including the epithelium, *Lamina propria mucosae* (**Supplementary Figures 6A-i**, **5C-i**), and within the intestinal crypts (**Supplementary Figures 6A-ii**, **5B-ii**).

Interestingly, Dkk*1*-expression was considerably lower in the crypt bottom compared to more superficial epithelial cells (**Supplementary Figures 6A-ii**, asterisk). Additionally, *Dkk1-*, *Dkk3-*, and *Krm2-*transcripts were examined within smooth muscle cells of the *Tunica muscularis* (**Supplementary Figures 6A-iii, C-iii, D-iii**). In the large intestine, *Dkk1*- and *Dkk3*-transcrips were expressed within the epithelium (**Supplementary Figures 6A-i, C-i**). Additionally, *Dkk3* exhibited an expression down to the base of the crypts, in the *Lamina propria mucosae* below the crypt region and within the *Lamina muscularis mucosae* (**Supplementary Figures 7C-ii**). *Dkk2* was weakly detectable in epithelial cells, at the crypt bottom as well as in smooth muscle cells of the *Tunica muscularis* of the small intestine (**Supplementary Figures 6B-iii**).

In the large intestine, *Dkk2*-mRNAs were mostly present within the superficial cells of the epithelium and less so in the crypts (**Supplementary Figures 7B-i-ii**). Moreover, weak expression of *Dkk4* was found in intestinal crypts of the small intestine (**Supplementary Figures 6E-i**), surrounding the crypt bottom. In contrast, in the large intestine Dkk4-mRNAs were mainly detected in epithelial cells outside the crypts (**Supplementary Figures 7E-i**) and in the *Lamina muscularis mucosae* (**Supplementary Figures 7E-ii**). Additionally, *Dkk4*-transcripts were also present within the *Tunica muscularis* in both gut regions (**Supplementary Figures 6E-ii**, **7E-iii**).

In the small intestine, *Krm1*-transcripts were mainly localized within crypts, and in the *Tela submucosa* and *Tunica muscularis* (**Supplementary Figures 6F-i-ii**). In the large intestine, *Krm1* was slightly expressed within some epithelial cells, however, predominantly localized at the crypt bottom, in the *Lamina propria mucosae* surrounding the crypts, as well as weakly within the *Tunica muscularis* (**Supplementary Figures 7D-i-ii**). *Krm2*-transcripts showed a broader presence than *Krm1*-transcripts, including the expression, throughout the villus and *Lamina propria mucosae*, the crypt bottom, as well as within the *Tunica muscularis* of the small intestine (**Supplementary Figures 6D-i-iii**). In large intestine, *Krm2-*expression was less intense, but also present within the epithelium including the crypt bottom and within the *Tunica muscularis* (**Supplementary Figures 7F-i-iii**).

### Expression of the Wnt-agonist R-Spondin in mouse intestine

**Figure 6:**
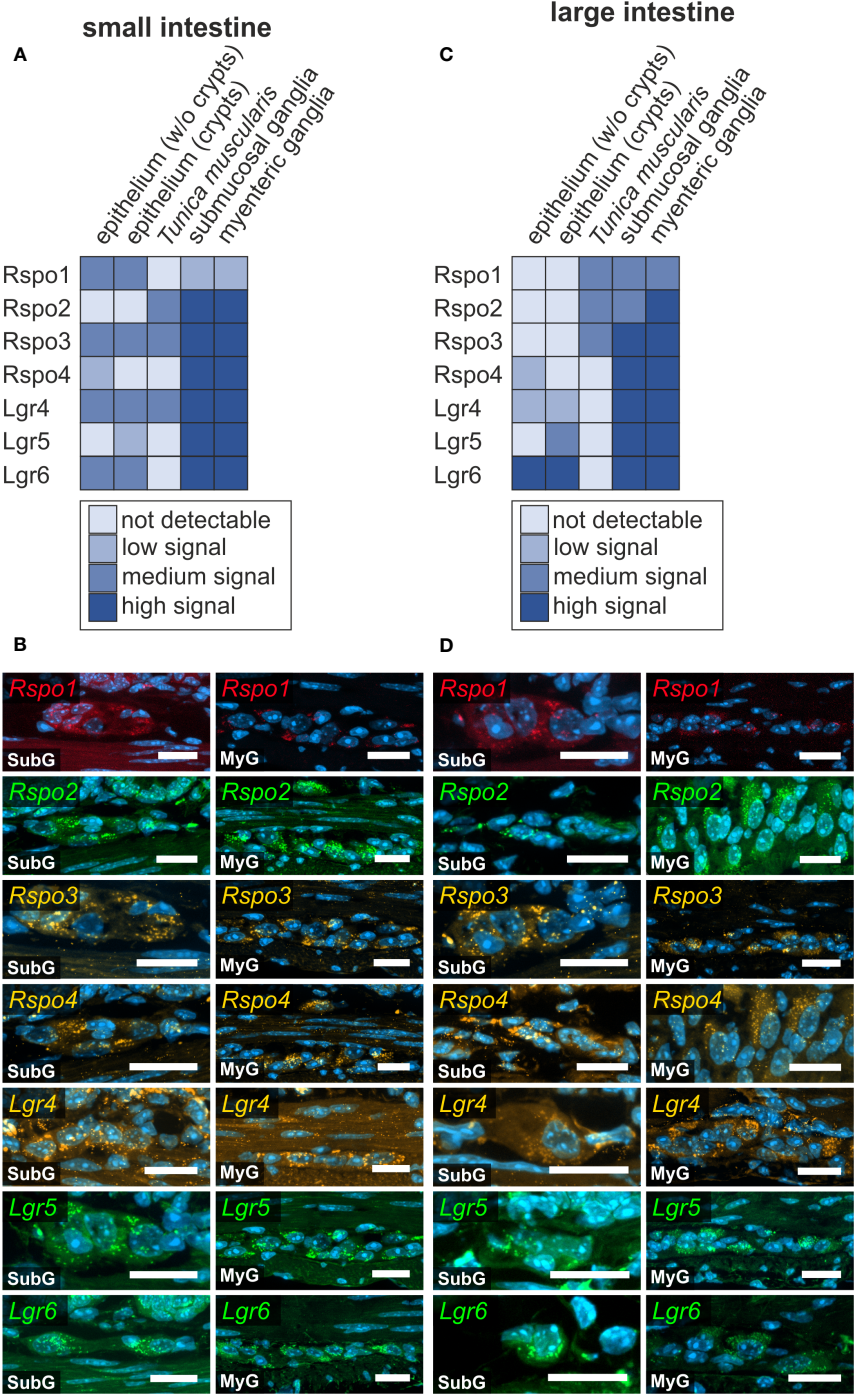
R-spondin-ligands and Lgr-receptors are present in the murine small and large intestine. **(A–D)**: *In situ* hybridization experiments showed *Rspo1-, Rspo2-, Rspo3-, Rspo4-, Lgr4-, Lgr5-, and Lgr6-*mRNA detection (colors as indicated) and the nuclear marker DAPI (blue) within submucosal (SubG) and myenteric plexus (MyG) of murine small **(A, B)** and large intestine **(C, D)** with different signal levels. Scale bars: 20 µm.

In cells with ongoing Wnt-signaling, R-Spondin proteins interact with their cognate receptors LGR4, LGR5, and LGR6 to inhibit ligase activity that would otherwise degrade Frizzled receptors, as part of a negative feedback mechanism. This in turn leads to accumulation of Frizzled receptors on the plasma membrane, thus sensitizing target cells towards Wnt ligands (40, 41). Here, we examined the expression of R-Spondin-ligands 1-4 *(Rspo1, Rspo2, Rspo3, Rspo4)*, and the relevant receptors *Lgr4*, *Lgr5, and Lgr6* in the murine ENS. We found that all RSPO-ligands were expressed in the submucosal as well as the myenteric plexus of the small (**Figures 6A, B**) and large intestine (**Figures 6C, D**). Yet, *Rspo2-, Rspo3-, Rspo4-*transcripts exhibited a more abundant and stronger expression than *Rspo1* (**Figures 6B, D**).

Moreover, Lgr4, Lgr5, and Lgr6 were strongly expressed in both enteric plexus of the small and large intestine. Intriguingly, whereas *Lgr5* and *Lgr6*-transcripts displayed a clear and distinguishable localization within neuronal perikarya compared to glial cell bodies or the ganglionic neuropil, *Lgr4*-transcripts showed a rather homogeneous expression throughout the different cells of the ganglia (**Figures 6A, B**). Furthermore, a similar expression pattern was observed in submucosal and myenteric ganglia of the large intestine (**Figures 6C, D**, **Supplementary Table 1**). Similar results were obtained from the single cell RNA sequencing dataset, however *Rspo4* transcripts were not found in the glial clusters (**Supplementary Table 2**).

R-Spondins and Lgr-receptors were also found in other layers of the gastrointestinal wall. We detected a considerable expression of *Rspo1* in within the epithelium of the small intestine (**Supplementary Figure 8A**), however, with no expression in the large intestine. *Rspo2-*expression was not detectable in small and large intestine samples. Moreover, *Rspo3-*mRNAs were found in the small intestine particularly within the *Lamina propria mucosae* (**Supplementary Figure 8B**, arrowhead) and in epithelial cells at the crypt bottom (**Supplementary Figure 8B**, arrow). Yet, *Rspo3* was not detectable in the large intestine. *Rspo4* was expressed within cells of the *Lamina propria mucosae* (**Supplementary Figure 8C**, arrow) and with lower expression intensity within the epithelium of the small intestine (**Supplementary Figure 8C**, arrowhead). In the large intestine, Rspo4 was restricted in its expression to the epithelium especially outside the crypts (**Supplementary Figure 9A**, arrowhead).

In the small intestine, a high expression of the corresponding receptor *Lgr4* was observed throughout the entire epithelium (**Supplementary Figures 8D-i**, arrowhead) and within the *Lamina propria mucosae* (**Supplementary Figures 8D-i**, arrow). In the large intestine, *Lgr4*-transcripts were found within the epithelium (**Supplementary Figures 9B-i**), as well as throughout the crypts (**Supplementary Figures 9B-ii**, arrow). However, we found a population of cells at the bottom of the crypt that clearly lacked the expression of *Lgr4* (**Supplementary Figures 9B-ii**, arrowhead). In contrast, *Lgr5*-mRNA was restricted to a subpopulation of epithelial cells at the crypt bottom as well as to cells of the *Lamina propria mucosae* in close vicinity to the crypt bottoms both, within the small (**Supplementary Figure 8E**, arrowhead) and large intestine (**Supplementary Figure 9C**, arrowhead). *Lgr6* was occasionally found in cells of the epithelium (**Supplementary Figures 8F-i**, arrowhead), and in a subset of cells located at the crypt bottom (**Supplementary Figures 8F-ii**, arrow), as well as within the *Lamina propria mucosae* of the small intestine (**Supplementary Figures 8F-i**, asterisks). In the large intestine, *Lgr6*-transcripts were found in epithelial cells (**Supplementary Figures 9D-i-ii**, arrowhead), within the *Lamina propria mucosae* (**Supplementary Figures 9D-ii**, asterisk), however not at the crypt bottom (**Supplementary Figures 9D-ii**, arrow).

To further contextualise the presence of Wnt-signaling related mRNAs under pathophysiological conditions, we re-evaluated a recently published glia-specific RiboTag-based RNA sequencing dataset of acute inflammation in a mouse model of post-operative ileus (21).

### Myenteric glial cells express Wnt-signaling related mRNAs in a model of post-operative ileus

A recently published study by Leuven and Schneider et al. describes enteric glial plasticity during acute intestinal inflammation in a post-operative ileus (POI) mouse model. In brief, they induced enteric glial reactivity, by a standardized intestinal manipulation (IM) procedure leading to POI. POI progression was classified into an early/immediate phase (3h after intestinal manipulation), inflammatory/manifestation phase (24h after intestinal manipulation), and recovery/resolution phase (72h after intestinal manipulation). With an established RiboTag-based approach (42), they selectively isolated transcribed mRNAs of hemagglutinin-labeled ribosomes of enteric glial cells of the *Tunica muscularis* of Sox10iCreERT2/Rpl22HA/+ mice within the relevant POI stages. We re-examined the resulted RNA-Seq dataset (22), thereby focusing on Wnt-signaling related mRNAs and found, that in total 31 Wnt-signaling related mRNAs were differently regulated during the designated timepoints. *Fzd2-*, *Krm1-*, *Rspo3-*, *Wnt5b-* and *Wnt6-*mRNA transcripts reached statistical significance during the early phase, 3h post IM (see **Supplementary Table 3**). However, their expression level normalized to the naive control population or were not detectable after 6h post IM. During the inflammatory phase, 24h post IM, *Dkk2* and *Fzd7* transcripts were upregulated, whereas *Dkk3* and *Sfrp1 (Secreted frizzled related protein 1)* transcripts were down regulated. After 72h post IM, *Dkk2*, *Sfrp1*, *Fzd2* and *Wnt5a mRNAs* expression levels were still significantly regulated.

In summary, by combining our *in situ* hybridization expression atlas with the enteric gliosis data shed light on a broader, arguably highly dynamic impact of canonical and non-canonical Wnt-signaling on the mature ENS and its cellular homeostasis in health and inflammation.

## Discussion

Histological evaluation of small lipid-modified morphogens, such as Wnt-molecules, poses considerable experimental challenges, one of which is the lack of reliable antibodies targeting Wnt-molecules in mammalian tissues (43). To cope with this problem, detecting mRNAs is an effective alternative, which also allows for a more specific identification of Wnt-secreting cells. Here, we applied the RNAscope HiPlex-assay, that uses a special amplification procedure to enable the detection of single mRNA transcripts, making it especially useful for the evaluation of tightly regulated signaling cascades with low abundant mRNA molecules at the cellular level (44). In this study, we present an overview of the expression pattern of 45 relevant Wnt-signaling components within the enteric nervous system, thereby closing a gap of knowledge about the role of Wnt-signaling in tissue homeostasis of the gastrointestinal tract. Our results are partly in concert with and thus substantiate previously published single cell RNA-sequencing analyses of the juvenile murine ENS of the small intestine (37), thereby adding a valuable special component to big data gene expression studies. Nevertheless, we want to point out that cross-interpretation between different studies should always be carried out carefully taking diverse experimental approaches (e.g., sample acquisition, cell isolation, age of animal) and varying sensitivity in the detection of target sequences into account.

### Source of Wnt-ligands in the ENS

Prior to secretion, newly synthesized Wnt-ligands undergo various posttranslational modifications in the endoplasmic reticulum (ER) of Wnt-producing cells. These modifications are indispensable for both, intracellular trafficking by the transmembrane protein Wntless (Wls) via the Golgi apparatus to the plasma membrane (i.e., Wnt-ligand secretion), and Wnt-receptor recognition by the target cell. After Wnt-ligand secretion, Wls is endocytosed and recycled to fulfil its trafficking function again (39). Wls indirectly plays an important role for ENS-development, as it is expressed during early embryogenesis at the mid-hindbrain boundary and mediates the secretion of Wnt1, which in turn induces neural crest formation (45). Furthermore, in humans loss of Wls is associated with severe multiorgan defects (46) and epithelial colorectal cancer (47), whereby the latter underlines its importance in tissue homeostasis.

We observed that Wls-transcripts are expressed throughout the cell bodies of submucosal and myenteric plexus cells in the small intestine and were less abundant in the large intestine. Together with previous reports (47, 48), our data indicate that the Wnt-producing cells are thus present within the adult enteric nervous system, suggesting an underlying, yet to be uncovered role in postnatal ENS tissue homeostasis.

Moreover, we found that the mRNA of Wnt ligands, such as Wnt1, Wnt2, or Wnt2b was primarily located in enteric perikarya rather than the neuropil or other intraganglionic cells, strongly indicating that not only glial cells but also enteric neurons themselves could serve as sources of Wnt ligands in the ENS. The expression of *Wnt1* mRNA by adult enteric neurons is particularly intriguing as Cre-lox mediated fluorescent reporter mouse strains widely used in the field make use of a Cre-recombinase under the control of a Wnt1-promotor (e.g., 129S4.Cg-E2f1Tg(Wnt1-cre)2Sor/J, The Jackson Laboratory, strain #022137). If crossbred with mice carrying floxed fluorescent reporter proteins, these strains are particularly useful in visualizing the ENS, as all neural crest derivatives (i.e., the ENS in the gut) initiate fluorescent protein expression during Wnt1-dependent neural crest induction. The re-occurrence of Wnt1-mRNA in adult enteric neurons, however, suggest that also Cre-recombinases are expressed at adult stages. It is therefore conceivable that mature enteric neurons would go through repetitive recombination events, if the floxed parent strain uses LoxP-sites that are oriented in opposite direction to each other in order to introduce an inversion of the floxed target gene sequence (see also (49) for recent review on Cre-LoxP strategies). Therefore, beyond its physiological implications, our data point out methodological caveats when planning reporter and/or knock-out experiments using Wnt1-Cre dependent mouse models.

### Transport of Wnt-ligands and the extracellular matrix of the ENS

As a result of the posttranslational modifications, Wnt-ligands are hydrophobic and are thus unlikely to diffuse freely within the aqueous extracellular milieu to reach the target cell (for more details on posttranslational modifications, see the recent review by Mehta et al. (50). Yet, the underlying mechanism by which Wnt-ligands are dispersed is hardly characterized in mammals compared to studies in Drosophila (51) and Xenopus (52). Intriguingly, there are implications suggesting that posttranslational modifications (53) enable interactions with extracellular matrix components like heparin sulfate proteoglycans (HSPGs), which are also present on the cell-surface of Wnt-receiving cells (54). This lead to a diffusion model, where Wnt-ligands are transported in a bucket brigade manner by repeated association and dissociation with HSPGs on cell membranes [see also (55)]. Still, there is hardly any data available on secretion and transport processes of Wnt-ligands in the adult enteric nervous system. Nonetheless, immunohistochemical analysis of two heparin sulfate proteoglycans, namely collagen 18 and agrin, in chicken (36) as well as in rat and guniea pig (56) showed a strong expression surrounding enteric ganglia, thus, rendering HSPG-mediated extracellular/extra-ganglionic shuttling of Wnt-ligands plausible. In addition, extracellular matrix interaction of Wnt-ligands secreted from surrounding glia cells (20), myofibroblasts, pacemaker cells, fibroblasts, smooth muscle cells or macrophages (57) might influence adult ENS-homeostasis, as well. In this context, especially the role of resident macrophages of the muscularis externa has been recently shown to have a profound impact on early postnatal development of the ENS (17).

### Intracellular Wnt signaling in the target cell

After reaching a target cell, Wnt-ligands activate either β-catenin-dependent, canonical (classical agonists are Wnt1, Wnt3a or Wnt8a) or β-catenin-independent, non-canonical (Wnt4, Wnt5a or Wnt11) signaling cascades. However, it is noteworthy that the signaling output not only depends on the particular Wnt-family member, but is largely influenced by the cellular context and the combination of receptors expressed by the target cell (58). Thus, three core pathways have emerged as the main transducers of Wnt-signaling: (1) The Wnt/βcatenin signaling is of particular importance for cell proliferation. It is triggered by interaction between the canonical Wnt-ligands and a transmembrane receptor complex that is formed by the seven-pass transmembrane receptors of the Frizzled family (Fzd1-10), and the co-receptor low-density lipoprotein receptor-related protein 5 or 6 (LRP5/6) (59).

Among the non-canonical pathways (2), the Wnt/Planar cell polarity (PCP) pathway, employs a signaling cascade involving the small GTPases Rho and Rac and c-Jun N-terminal kinase prominently regulating remodeling of the cytoskeleton and cell differentiation processes (60). In contrast, the non-canonical (3) Wnt/calcium pathway triggers the release of intracellular Ca^2+^ and the activation of Ca^2+^-dependent kinases thereby controlling cell motility (61). In this regard our studies demonstrated that the canonical Wnt signaling pathway is inactivated between the transition from proliferation to differentiation in the *in vitro* model system of postnatally-derived murine ENS-progenitors (62). Moreover, pharmacological activation of canonical Wnt-signaling increases the proliferative capacity of postnatal ENS-progenitors from rodent models and human infants *in vitro* and successively leads to a higher yield of newly generated neurons (28). It is conceivable, that canonical Wnt signaling, together with the non-canonical Wnt pathways, shapes the cellular homeostasis and cytoarchitecture of the adult ganglionated plexus *in vivo*, as well.

### Fzd-Wnt interactions in the ENS

Despite the apodictic categorization of Wnt-ligands as canonical and non-canonical agonists, mapping a conclusive Fzd-Wnt interactome still is a challenge for the mammalian system, due to high cross-reactivity of individual Wnt-ligands with Fzd subtypes (63). The 10 mammalian Fzd-receptors can be divided into four discrete classes of receptors based on their structural homology. Hereby, Fzd-receptors within each class share between 50% (Fzd3 and Fzd6) and 97% (Fzd1, Fzd2 and Fzd7) amino acid identity (64, 65). In mammals, Frizzled receptors have been implicated in a variety of developmental processes of the central nervous system including neural tube closure (66), axonal outgrowth and guidance of major fiber tracts (67–69), or mediating neurogenic processes in the developing neural tube (70). However, in the adult, misexpression of Fzd receptors is often associated with development and progression of diverse cancers underlining the importance of Wnt-signaling in tissue homeostasis [reviewed in detail by Phesse et al. (71)].

Particularly, Fzd7 is known to activate non-canonical as well as canonical Wnt-signaling in various tissues during development, homeostasis, and disease in several species including humans (72). For instance, the interaction of Fzd7 with Wnt6 induces epithelialisation of the somite’s via canonical signaling cascade (73), and its interplay with Wnt11 orchestrates neural crest cell migration via non-canonical PCP and Ca^++^ signaling (74). In the adult intestine, Fzd7 is known to be expressed by epithelial stem cells (26) and Flanagan et al. observed an impaired capacity of these cells to regenerate the intestinal epithelium of Fzd7-knockout mice (75). Additionally, the latter study reported on the binding capacity of Fzd7 for the canonical Wnt-ligands Wnt3a as well as Wnt2b (75). These ligands are indispensable for ongoing canonical Wnt-signaling at the stem cell niche at the crypt base (76) and thus are essential to maintain the pool of cycling intestinal stem cells (77, 78). Hence, ectopic expression of Fzd7 has a profound role in tumorigenesis and is critical for the survival, invasion, and metastatic capabilities of colorectal cancer (79, 80). Comparable to the observations by Flanagan et al., we detected *Fzd7*-expressing cells within the crypts of small and large intestine. In addition, we found a prominent expression of the *Fzd7*-mRNA within submucosal and myenteric ganglia of adult mice. Interestingly, *Wnt3a* and *Wnt2b* were also expressed within submucosal and myenteric plexus cells.

Beyond that, in the small intestine, we detected the expression of supposedly non-canonical Wnt-ligands *Wnt5a, Wnt7a, Wnt8b* and *Wnt11* within the ENS, whereby the latter was not expressed in large intestine samples. It should be mentioned, that although Wnt5a is an important agonist of non-canonical Wnt/Ca^++^-signaling (81), it also activates canonical Wnt-signaling in the presence of the Lrp5- and Fzd4-receptors (81, 82). Interestingly, in the current study we found Fzd4-expression in submucosal and myenteric plexus of small and large intestine. This is in accordance with previous studies showing, that the Wnt-receptor Fzd4 is expressed on protein level in the human colonic myenteric plexus in Hirschsprung patients (83) and moreover is useful to purify ENS-progenitor cells (27). Further, some Wnt-ligands have been shown to play an important role in the development of the avian intestine. Nagy and colleagues showed, that *Wnt11* is expressed in the ceca during hindgut ENS formation and highlighted its importance as an activator of non-canonical Wnt signaling in regulating neural crest cell differentiation (24). Furthermore, data gathered from transcriptome profiling of the widely used *in vitro* model system of postnatally derived and purified murine ENS-progenitors, suggests that mesenchymal cells express neural stem cell supportive ligands including *Wnt5A* and *Wnt11.* Intriguingly, corresponding receptors such as *Fzd1, Fzd3, Fzd6*, and *Fzd7* were expressed by neural cells in the same study (31).

### Fine tuning of Wnt-signaling activity by Dickkopf and R-Spondin ligands

Due to its pivotal role in regulating proliferation and differentiation in various organs, Wnt-signaling is tightly regulated. This is achieved not only through the divergent expression of ligands and receptors with partially overlapping function, but also through the secretion of various co-activators and inhibitors that allow for precise fine-tuning of Wnt-activity (84). The prominent group of Wnt-signaling antagonists namely Dickkopf-related proteins are an evolutionary conserved gene family of four glycoproteins (DKK1-4) (85). These proteins are involved in morphogenesis in diverse tissue types in vertebrates (85), and further control a variety of cellular/context-dependent functions, like cell proliferation (86) and differentiation processes (87–89), cell survival and programmed cell death (90). Particularly, DKK1, 3 and 4 interfere with canonical Wnt-signaling by binding LRP5/6 and forming a tertiary complex with their respective receptors Kremen 1 and 2 (Krm1-2), which induce the endocytosis of the essential LRP5/6 receptors (91). Our group has recently published results about the ambivalent function of the Wnt-antagonist DKK1 on murine and human ENS-progenitor cells (29), suggesting a multidimensional regulatory network of Wnt-signaling components in the enteric nervous system. In the current study, we further substantiated our previous findings on DKK expression in the ENS by showing the expression of DKK-ligands and Kremen-receptors in the submucosal and myenteric plexus of the murine small and large intestine.

The regulatory network of Wnt-signaling is completed by the R-spondin protein family (roof plate–specific spondin), which comprises four members (RSPO1-4). These are thought to augment ongoing Wnt-signaling and were first described as secreted activators of canonical Wnt-signaling, and potent mitogens within the stem cell niche of the intestinal epithelium (92, 93). Within this stem-cell niche R-Spondins actively cooperate with Wnt-ligands, such as Wnt3a, Wnt6, and Wnt9 expressed by Paneth cells or the surrounding mesenchyme (94). By binding to the Leucine-rich repeat-containing G-protein coupled receptors 4-6 (LGR4-6), degradation of Frizzled-receptors is inhibited, thus increasing the sensitivity of these target-cells for Wnt-activation (95), enabling the continuous regeneration of the intestinal epithelium (40, 41). Moreover, the corresponding signaling receptor of R-Spondins Lgr5, is an established stem cell marker in adult epithelial stem cell niches (96, 97). Interestingly, unlike in epithelial stem cell niches, Yu and colleagues reported about LGR5-expression in terminally differentiated neurons, but not in the stem cell population of the olfactory bulb (98). Moreover, LGR5- and RSPOs-expression was observed during the WNT-dependent maturation processes in cerebellar granule neurons (99). In this context, we found several R-Spondin ligands as well as corresponding receptors expressed within submucosal and myenteric ganglia of small and large intestine. Especially, LGR5 and LGR6 exhibited a clear localization within enteric neurons. Moreover, intriguingly, the study by Stavely et al. reported R-Spondin 1 and 3 expression in mesenchymal cells and corresponding LGR4 receptor expression by neural cells *in vitro* (31), thus emphasizing a yet undiscovered role of Wnt-RSPO-LGR signaling in postnatal homeostasis.

Just like Wnt-ligands, Dickkopf- as well as R-Spondin-ligands share posttranslational modifications, that enable interactions with heparin sulfate proteoglycans thereby facilitating extracellular transportation (34, 35, 100). Interestingly, mechanical signals mediated via the extracellular matrix have been reported to influence Wnt-ligand and Wnt-antagonist secretion (101, 102) as well as Fzd-receptor expression (103), resulting in pro-proliferative or pro-differentiation signals. As the adult ENS is constantly motile, changes in the physical property of the surrounding extracellular matrix initiate, not only reflex activity by mechanosensitive enteric neurons (104), but might also influence homeostatic processes in the postnatal ENS *in vivo*, conceivably by Wnt-signaling components linked to the extracellular matrix.

### Wnt-signaling and intestinal immunity

The enteric nervous system and the gut-resident immune system are in constant reciprocal interaction to maintain and modulate intestinal immunity. Conversely, the intestinal immune system influences maintenance of ENS integrity in health and disease and thus shapes ENS structure and function (17). Yet, the pathways of this neuro-immune interaction are still poorly elucidated, and little is known about respective impact of direct neuron-to-immune cell contacts and indirect interactions via intermediary cells, such as enteric glial cells [reviewed in detail by Seguella and Gulbransen (105)].

Although there is hardly any data available on the ENS-derived Wnt-signaling niche, recently published studies suggest, that Wnt-signaling might enable bidirectional communication between enteric glial cells, epithelial cells and immune cells in intestinal immunity. As mentioned above, Baghdadi et al. showed that Wnt ligands, particularly Wnt2, -4, -5a, and -6, are secreted by a subpopulation of type III glial cells in the *Lamina propria mucosae* and thereby influence epithelial regeneration and barrier function in mice. Interestingly, their results indicate that enteric glial-derived Wnts are dispensable for proper crypt homeostasis under healthy conditions arguably due to functional compensation by Wnts from other stromal sources. However, Wnt ligands from GFAP positive enteric glial cells were important for the high regenerative capacity necessary for the recovery from acute and chronic colitis (20). Further, in their recent study Leuven and Schneider et al. highlighted the plasticity of enteric glial cells during acute inflammation using a post-operative ileus (POI) mouse model (22). By glial specific RiboTag based RNA sequencing, they discovered differentially regulated gene expression profiles during the early/immediate phase (3h after surgery), inflammatory/manifestation phase (24h after surgery), and recovery/resolution phase (72h after surgery). Enteric glial cells switched to an acute immunoreactive phenotype, thereby expressing chemotactic factors, and shaping their microenvironment. At the disease peak, 24h after intestinal manipulation, glial cell reactivity was hallmarked by a migratory and highly proliferative phenotype before declining to a resolution state. Intriguingly, the study showed increased expression of the proliferation-marker Ki67 in a glial subpopulation. Additionally, revisiting their datasets, we found several Wnt-related mRNAs regulated. Interestingly, the presence of the proliferative gilal phenotype coincidences with the downregulation of contra-proliferative Wnt-signals like *Dkk* ligands or *Sfrp1* mRNA. This could indicate a link between enteric gliosis/acute inflammation and our previous works on Wnt-dependent cell-expansion in enteric neural progenitor cells *in vitro* (28, 29). Additionally, we found that pro-inflammatory *Wnt5a* expression was steadily decreasing over all tested timepoints reaching statistical significance at 72h post-surgery along with a detectable, yet not significant, expression of its inhibitor *Sfrp5* in the early phase. Since the Wnt5a/sFRP5 system is involved in the fine tuning of inflammatory responses in different tissues (106–108), these results might indicate that the enteric glial cells counteracts/limit inflammatory reactions via the Wnt-signaling pathway.

## Concluding remarks

Our high-throughput study demonstrated that, a plethora of Wnt-signaling components are expressed within the ENS, indicating a yet to be elucidated functional role of WNTs in ENS-homeostasis. Given the importance of the extracellular matrix for transportation as well as its supposed regulatory influence on secretion of Wnt-signaling components, suggests that further studies are needed to elucidate how morphogens are transported within the ENS. We are aware, that our study does not address the functional role of expressed mRNAs carried out by their translated proteins. Additionally, we want to emphasize, that a detailed cellular mapping of the gene expression patterns of selected genes using combined immunohistological analyses are needed to uncover the missing cell-type specific localization. Especially the lack of differentiability between immune cells like intraganglionic macrophages or leukocytes would be helpful to further stratify the immunological functions of Wnt-signaling related molecules.

However, this is beyond the scope of this work and up to future studies. Still, the presented data, bridges the existing gap of knowledge about the presences of Wnt-signaling components in the so far neglected ENS *in vivo*. Finally, together with recently published *in vitro* studies (28, 29), it highlights the importance of the multidimensional regulatory network of Wnt-signaling in the postnatal enteric nervous system that needs to be discovered.

## Data availability statement

The raw data supporting the conclusions of this article will be made available by the authors, without undue reservation.

## Ethics statement

The animal study was approved by Regierungspräsidium Tübingen, Landratsamt Tübingen, Veterinäramt, Tübingen, Germany. The study was conducted in accordance with the local legislation and institutional requirements.

## Author contributions

MS: Conceptualization, Data curation, Formal analysis, Investigation, Methodology, Software, Validation, Visualization, Writing – original draft. BH: Conceptualization, Data curation, Formal analysis, Funding acquisition, Visualization, Writing – review & editing. PN: Conceptualization, Data curation, Formal analysis, Funding acquisition, Investigation, Project administration, Supervision, Validation, Visualization, Writing – review & editing.

## Supplementary material

Supplementary Figure 1 Positive and negative controls. **(A)**: Representative micrographs show mRNA expression of the housekeeping genes GAPDH and HPRT in *Tunica mucosa*, *Tunica submucosa* and *Tunica muscularis* within murine small intestine (i) and large intestine (ii). In contrast, UBC expression was detected within the villi and the *Tunica muscularis* of small intestine (i+ii), as well as within the *Tunica muscularis* (iii) of the large intestine. Of note, all three housekeeping genes were expressed within the enteric nervous system. Scale bars: 40 µm. **(B)**: shows a representative negative control of murine large intestine. Scale bar: 100 µm. **(C)**: micrographs depict the non-expressed Wnt-ligands *Wnt7a*, *Wnt10b* and *Wnt11* in murine small intestine and *Wnt7a*, *Wnt9b* and *Wnt10b* in large intestine. Scale bars: 40µm.

Supplementary Figure 2 mRNA expression of Wntless and Wnt-ligands within *Tunica mucosa* and *Tunica muscularis* of murine small intestine. *Wnt2* **(A)**, *Wnt2b* **(B)**, *Wnt3a* **(C)**, *Wnt6* **(D)**, *Wnt7b* **(E)**, *Wnt8a* **(F)**, *Wnt8b* **(G)**, *Wnt9a* **(H)**, *Wnt9b* **(I)** and *Wls* (J) transcripts (colors as indicated) are found within different compartments of the intestinal wall (i-iii) of murine small intestine with different expression levels. Cell nuclei are stained for the nuclear marker DAPI (blue). Arrow as well as arrowheads point to positive cells. Scale bars: 40 µm.

Supplementary Figure 3 Presence of Wntless and Wnt-ligand transcripts within *Tunica mucosa* and *Tunica muscularis* of murine large intestine. *Wnt2* **(A)**, *Wnt2b* **(B)**, *Wnt3a* **(C)**, *Wnt6* **(D)**, *Wnt7b* **(E)**, *Wnt8a* **(F)**, *Wnt8b* **(G)**, *Wnt9a* **(H)** and *Wls* **(J)** transcripts (colors as indicated) are expressed within the intestinal wall (i-iii) of murine large intestine with different intensity levels. Cell nuclei are stained for the nuclear marker DAPI (blue). Arrow as well as arrowheads point to positive cells. **Scale bars: 40 µm**.

Supplementary Figure 4 Frizzled and Lrp receptors are expressed in different compartments of the murine small intestine. Detection of *Fzd1* **(A)**, *Fzd2* **(B)**, *Fzd4* **(C)**, *Fzd5* **(D)**, *Fzd6* **(E)**, *Fzd7* **(F)**, *Fzd8* **(G)**, *Fzd9* **(H)**, *Fzd10* **(I)**, *Lrp5* **(J)** and *Lrp6* **(K)** transcripts (colors as indicated) and the nuclear marker DAPI (blue) in different compartments (i-iii) of the small intestine. Arrow as well as arrowheads point to positive cells. Scale bars: 40 µm.

Supplementary Figure 5 Frizzled- and Lrp-receptor mRNAs are present within the murine large intestine. *In situ* hybridization experiments detected *Fzd1* **(A)**, *Fzd5* **(B)**, *Fzd6* **(C)**, *Fzd7* **(D)**, *Fzd9* **(E)**, *Lrp5* **(F)** and *Lrp6* **(G)** transcripts (colors as indicated) and the nuclear marker DAPI (blue) in different compartments (i-iii) of the murine large intestine. Scale bars: 40 µm.

Supplementary Figure 6 The Wnt antagonist Dickkopf and corresponding receptors Kremen are expressed in the murine small intestine. Micrographs depict expression of *Dkk1* **(A)**, *Dkk2* **(B)**, *Dkk3* **(C)**, *Krm2* **(D)**, *Dkk4* **(E)** and *Krm1* **(F)**, (colors as indicated) and the nuclear marker DAPI (blue) within the gastrointestinal wall (i-iii) of the murine small intestine. Asterisk indicates no expression at crypt bottom. Scale bars: 40 µm.

Supplementary Figure 7 Prominent Wnt-signaling antagonists are expressed in the murine intestine. Representative images show detection of *Dkk1* **(A)**, *Dkk2* **(B)**, *Dkk3* **(C)**, *Krm1* **(D)**, *Dkk4* **(E)** and *Krm2* **(F)**, (colors as indicated) and the nuclear marker DAPI (blue) within the gastrointestinal wall (i-iii) of the murine large intestine. Scale bars: 40 µm.

Supplementary Figure 8 R-spondin-ligands and Lgr-receptors are present in the murine small intestine. *In situ* hybridization experiments showed *Rspo1-* **(A)** *, Rspo3-* **(B)** *, Rspo4-* **(C)** *, Lgr4-* **(D)** *, Lgr5-* **(E)** *, and Lgr6* **(F)** *-*mRNA detection (colors as indicated) and the nuclear marker DAPI (blue) within different compartments of the murine small intestine. Arrows, arrowheads, and asterisk indicate respectively for the corresponding transcript expressing or non-expressing cells. Scale bars: 40 µm.

Supplementary Figure 9 R-spondin-ligands and Lgr-receptors are present in the murine large intestine. Representative images depict the expression of *Rspo4-* **(A)** *, Lgr4-* **(B)** *, Lgr5-* **(C)** *, and Lgr6* **(D)** *-*mRNAs and the nuclear marker DAPI (blue) within the gastrointestinal wall of the large intestine. Arrows, arrowheads, and asterisk indicate respectively for the corresponding transcript expressing or non-expressing cells. Scale bars: 40 µm.
