# Supplementary figures and images for: Spatial gene expression profile of Wnt-signaling components in the murine enteric nervous system
Source: Front Immunol. 2024 Jan 18;15:1302488. doi: 10.3389/fimmu.2024.1302488 (PMC10846065; doi:10.3389/fimmu.2024.1302488)

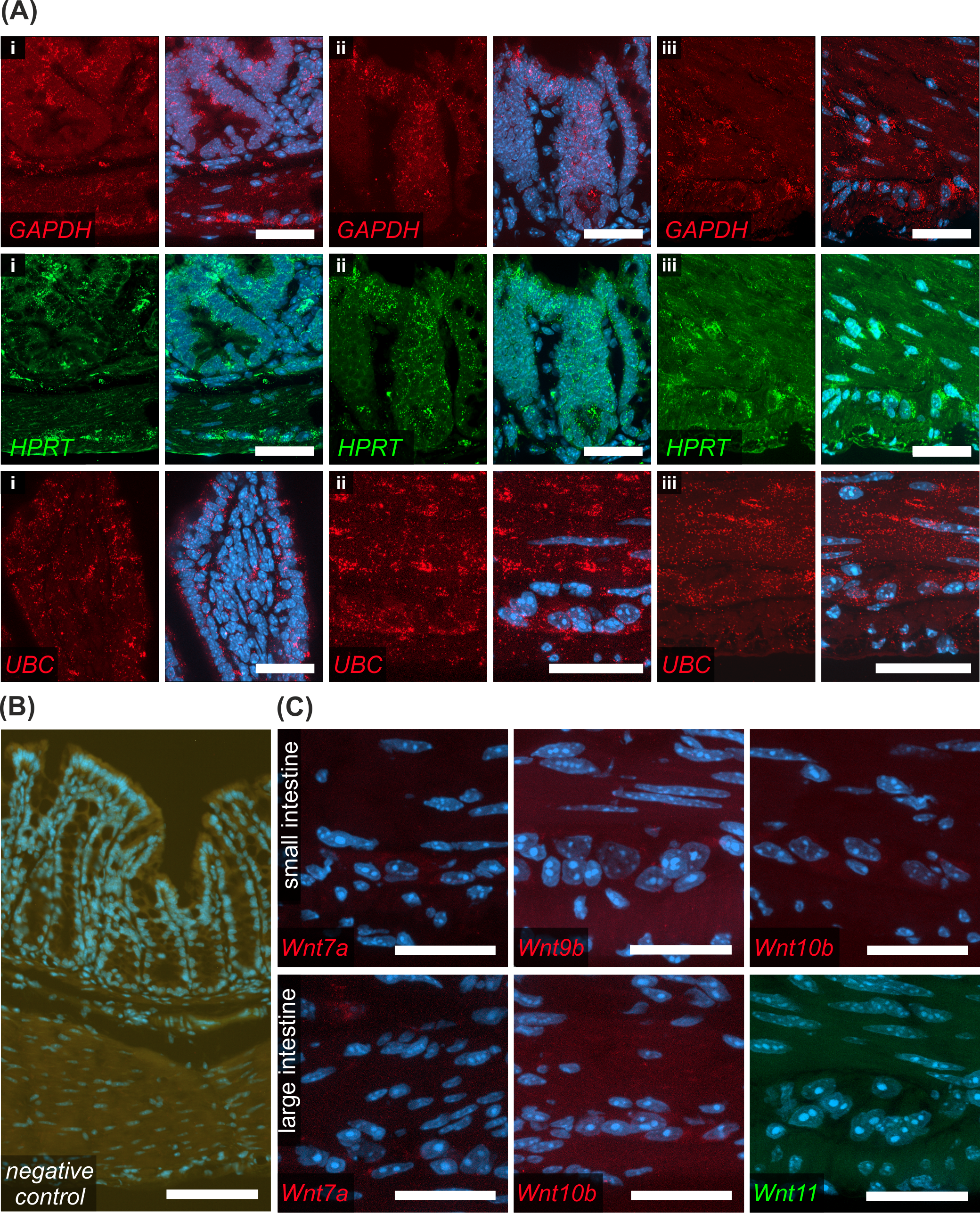

Supplement: Supplementary Figure 1 — Positive and negative controls. (A): Representative micrographs show mRNA expression of the housekeeping genes GAPDH and HPRT in Tunica mucosa, Tunica submucosa and Tunica muscularis within murine small intestine (i) and large intestine (ii). In contrast, UBC expression was detected within the villi and the Tunica muscularis of small intestine (i+ii), as well as within the Tunica muscularis (iii) of the large intestine. Of note, all three housekeeping genes were expressed within the enteric nervous system. Scale bars: 40 µm. (B): shows a representative negative control of murine large intestine. Scale bar: 100 µm. (C): micrographs depict the non-expressed Wnt-ligands Wnt7a, Wnt10b and Wnt11 in murine small intestine and Wnt7a, Wnt9b and Wnt10b in large intestine. Scale bars: 40µm. [file Image_1.tif]

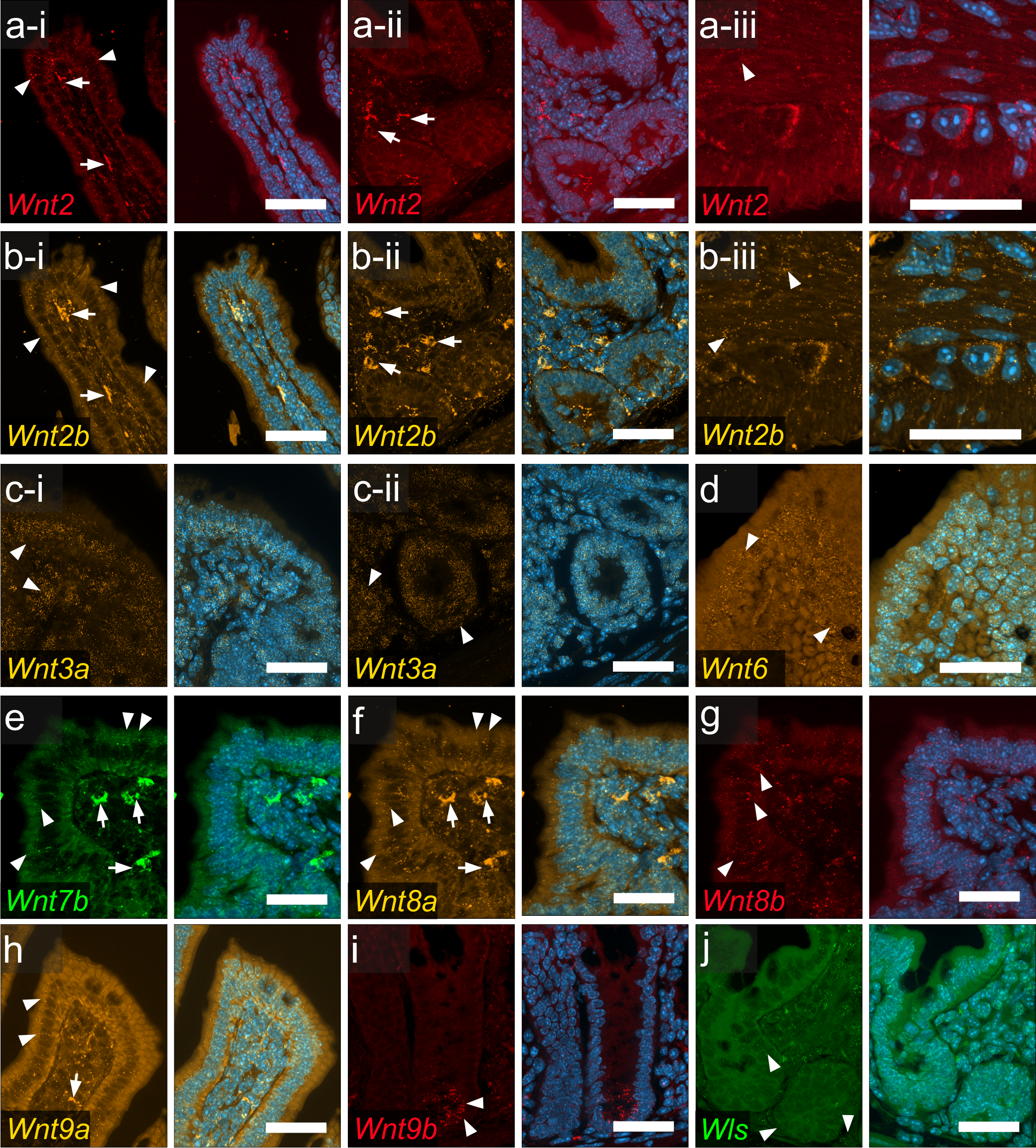

Supplement: Supplementary Figure 2 — mRNA expression of Wntless and Wnt-ligands within Tunica mucosa and Tunica muscularis of murine small intestine. Wnt2 (A), Wnt2b (B), Wnt3a (C), Wnt6 (D), Wnt7b (E), Wnt8a (F), Wnt8b (G), Wnt9a (H), Wnt9b (I) and Wls (J) transcripts (colors as indicated) are found within different compartments of the intestinal wall (i-iii) of murine small intestine with different expression levels. Cell nuclei are stained for the nuclear marker DAPI (blue). Arrow as well as arrowheads point to positive cells. Scale bars: 40 µm. [file Image_2.tif]

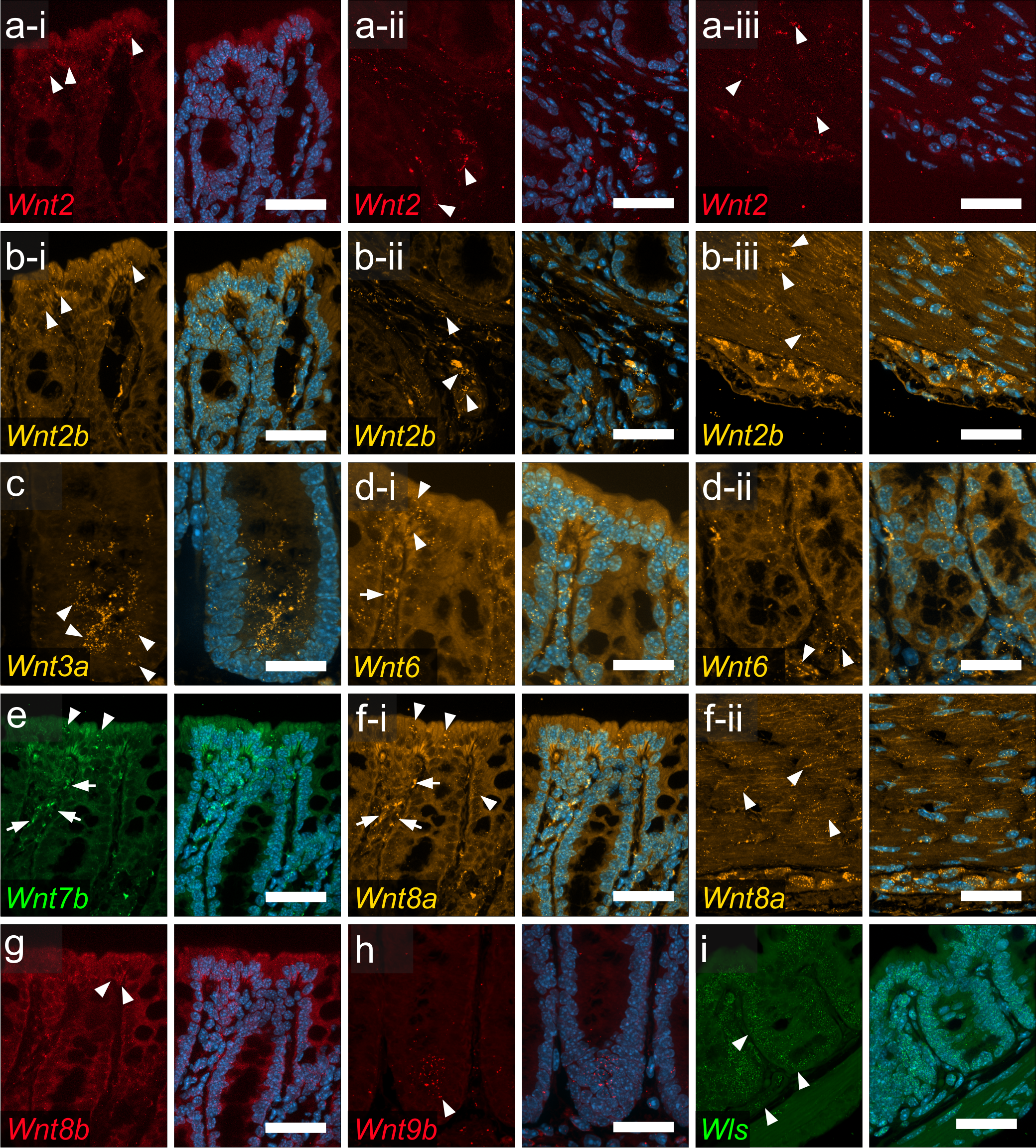

Supplement: Supplementary Figure 3 — Presence of Wntless and Wnt-ligand transcripts within Tunica mucosa and Tunica muscularis of murine large intestine. Wnt2 (A), Wnt2b (B), Wnt3a (C), Wnt6 (D), Wnt7b (E), Wnt8a (F), Wnt8b (G), Wnt9a (H) and Wls (J) transcripts (colors as indicated) are expressed within the intestinal wall (i-iii) of murine large intestine with different intensity levels. Cell nuclei are stained for the nuclear marker DAPI (blue). Arrow as well as arrowheads point to positive cells. Scale bars: 40 µm. [file Image_3.tif]

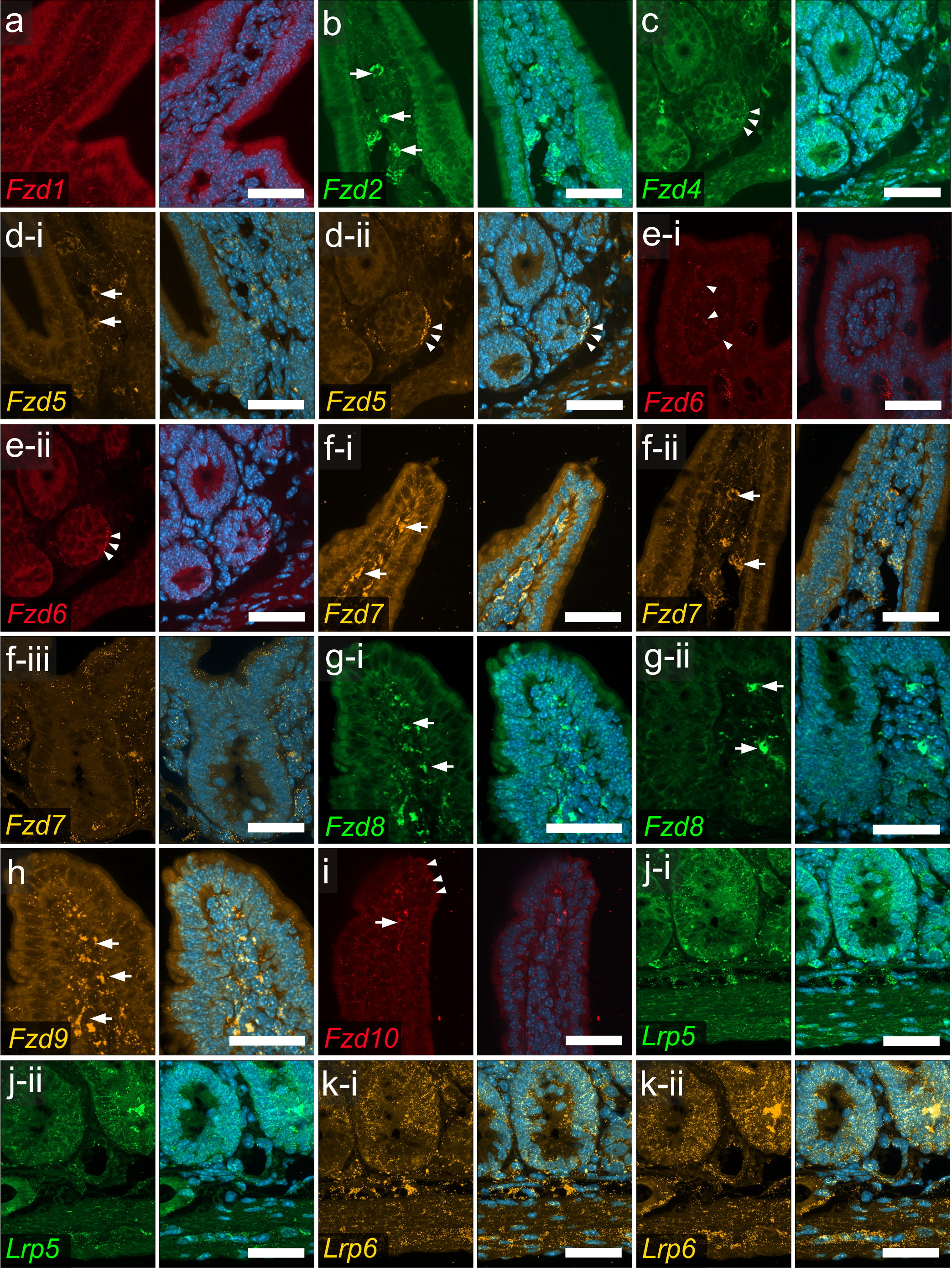

Supplement: Supplementary Figure 4 — Frizzled and Lrp receptors are expressed in different compartments of the murine small intestine. Detection of Fzd1 (A), Fzd2 (B), Fzd4 (C), Fzd5 (D), Fzd6 (E), Fzd7 (F), Fzd8 (G), Fzd9 (H), Fzd10 (I), Lrp5 (J) and Lrp6 (K) transcripts (colors as indicated) and the nuclear marker DAPI (blue) in different compartments (i-iii) of the small intestine. Arrow as well as arrowheads point to positive cells. Scale bars: 40 µm. [file Image_4.tif]

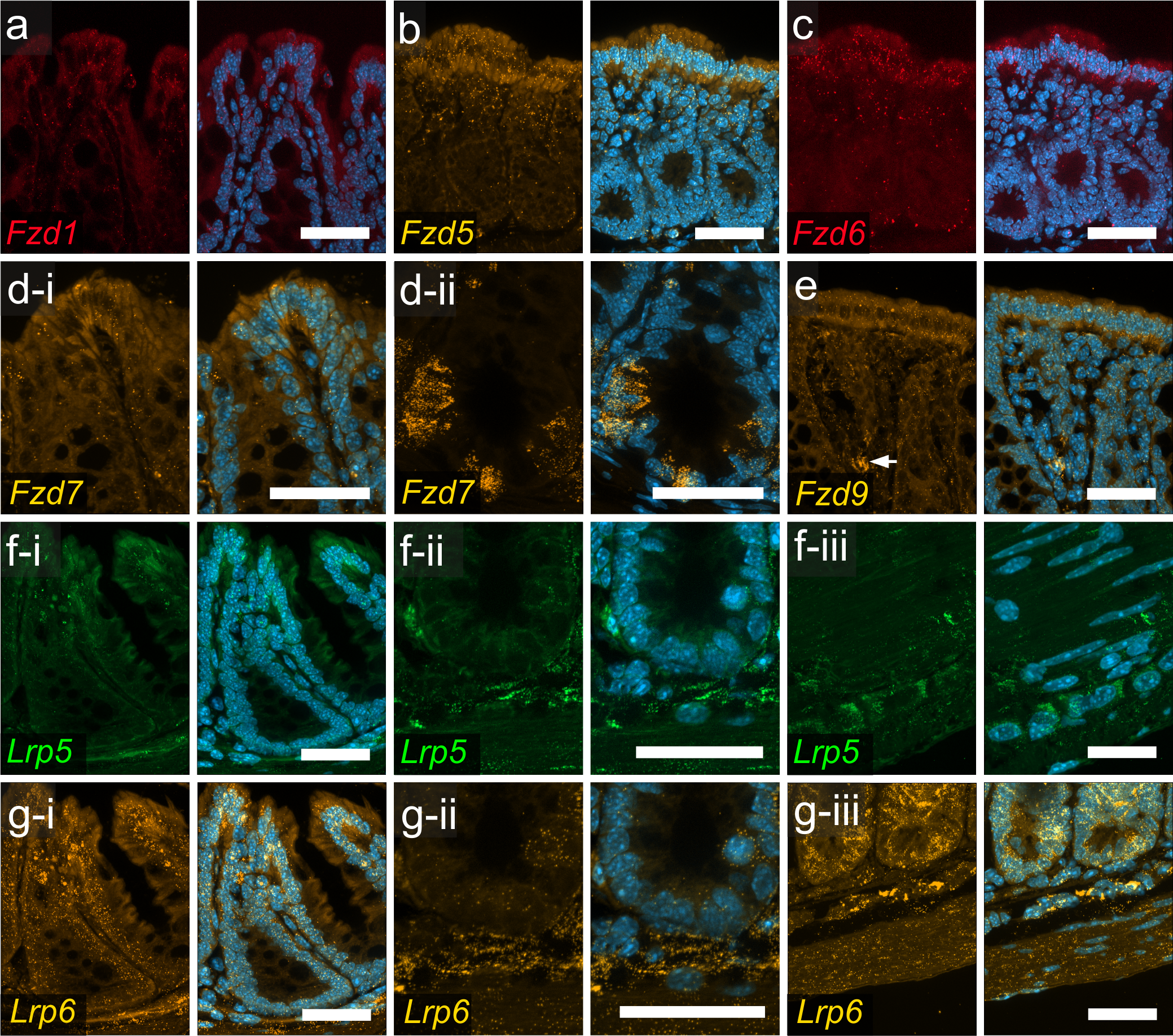

Supplement: Supplementary Figure 5 — Frizzled- and Lrp-receptor mRNAs are present within the murine large intestine. In situ hybridization experiments detected Fzd1 (A), Fzd5 (B), Fzd6 (C), Fzd7 (D), Fzd9 (E), Lrp5 (F) and Lrp6 (G) transcripts (colors as indicated) and the nuclear marker DAPI (blue) in different compartments (i-iii) of the murine large intestine. Scale bars: 40 µm. [file Image_5.tif]

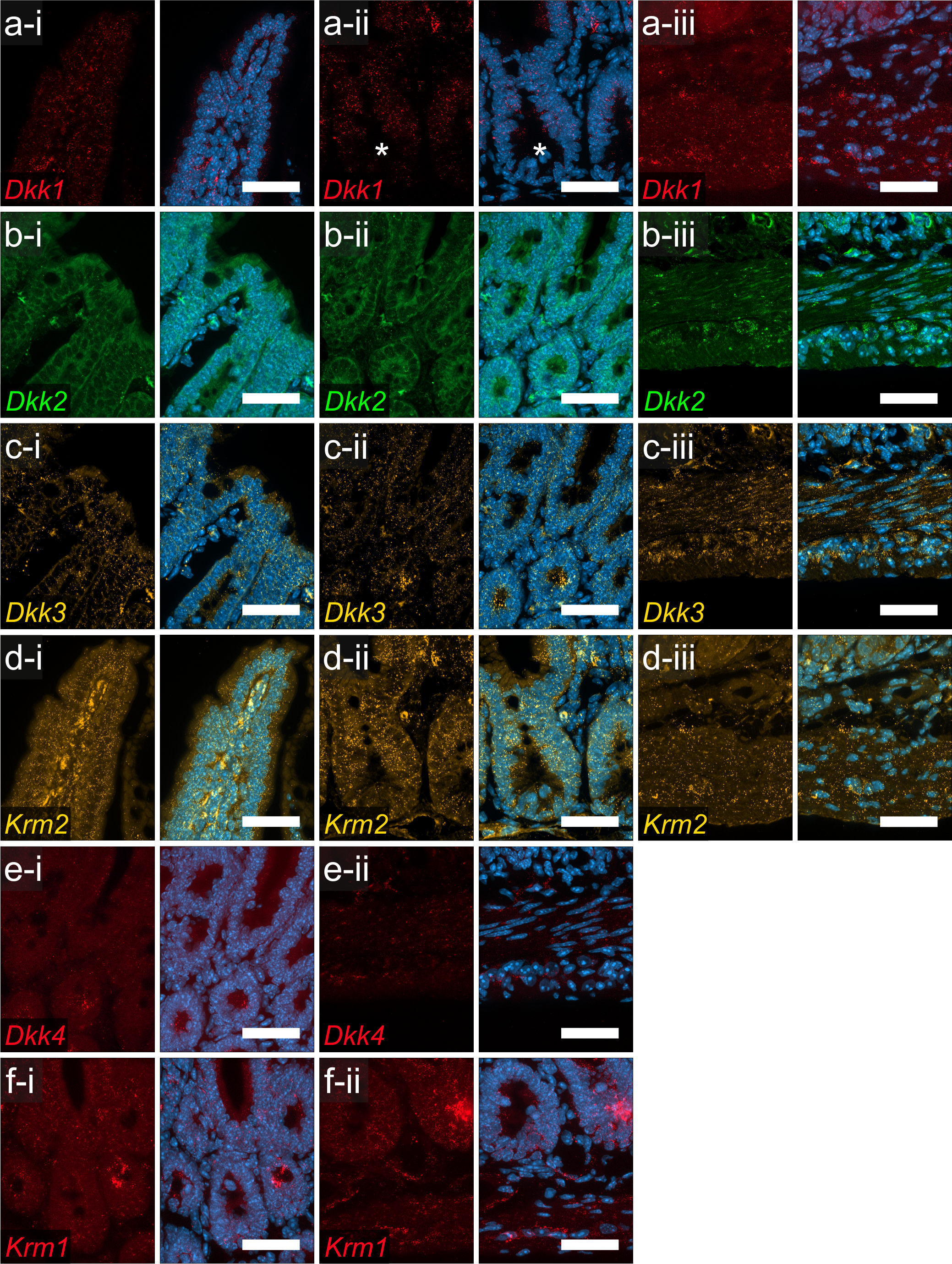

Supplement: Supplementary Figure 6 — The Wnt antagonist Dickkopf and corresponding receptors Kremen are expressed in the murine small intestine. Micrographs depict expression of Dkk1 (A), Dkk2 (B), Dkk3 (C), Krm2 (D), Dkk4 (E) and Krm1 (F), (colors as indicated) and the nuclear marker DAPI (blue) within the gastrointestinal wall (i-iii) of the murine small intestine. Asterisk indicates no expression at crypt bottom. Scale bars: 40 µm. [file Image_6.tif]

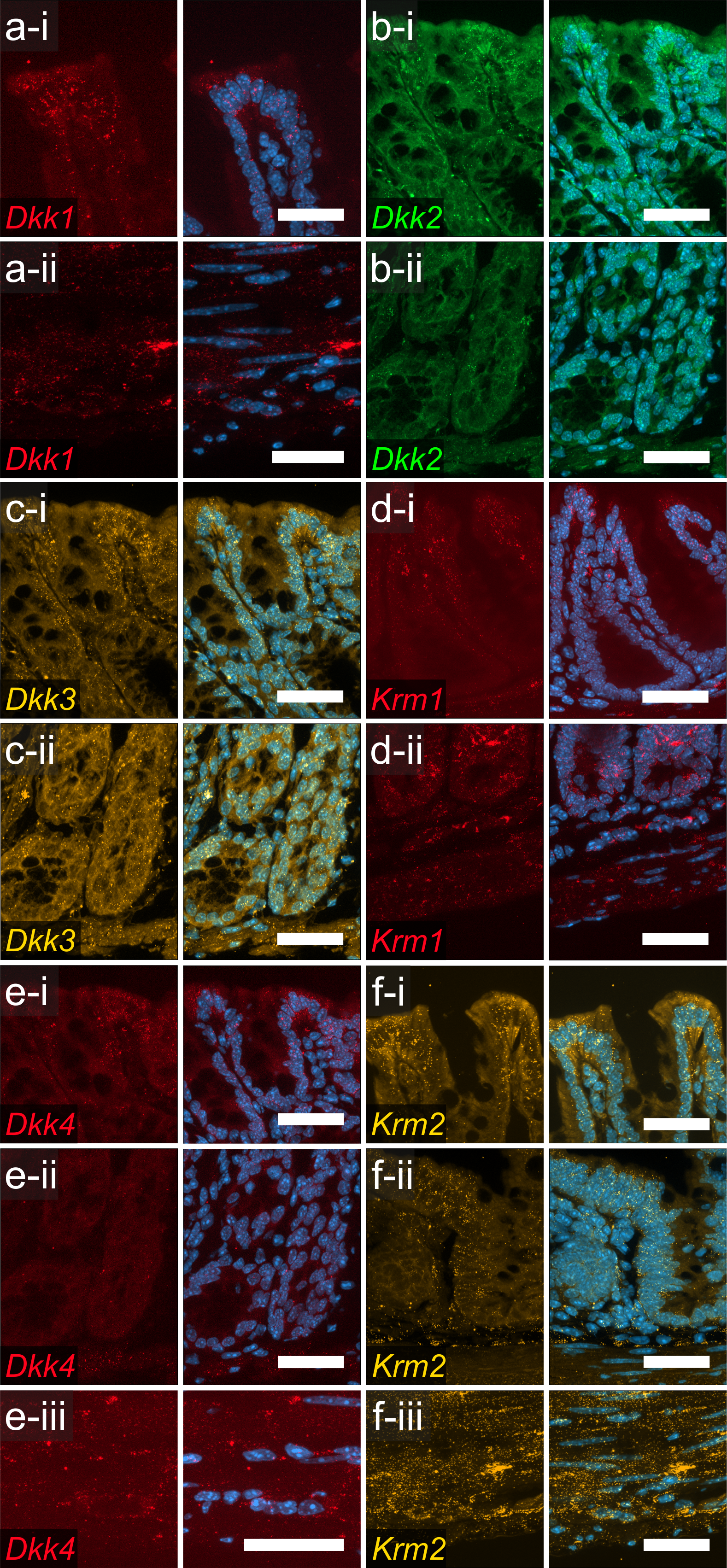

Supplement: Supplementary Figure 7 — Prominent Wnt-signaling antagonists are expressed in the murine intestine. Representative images show detection of Dkk1 (A), Dkk2 (B), Dkk3 (C), Krm1 (D), Dkk4 (E) and Krm2 (F), (colors as indicated) and the nuclear marker DAPI (blue) within the gastrointestinal wall (i-iii) of the murine large intestine. Scale bars: 40 µm. [file Image_7.tif]

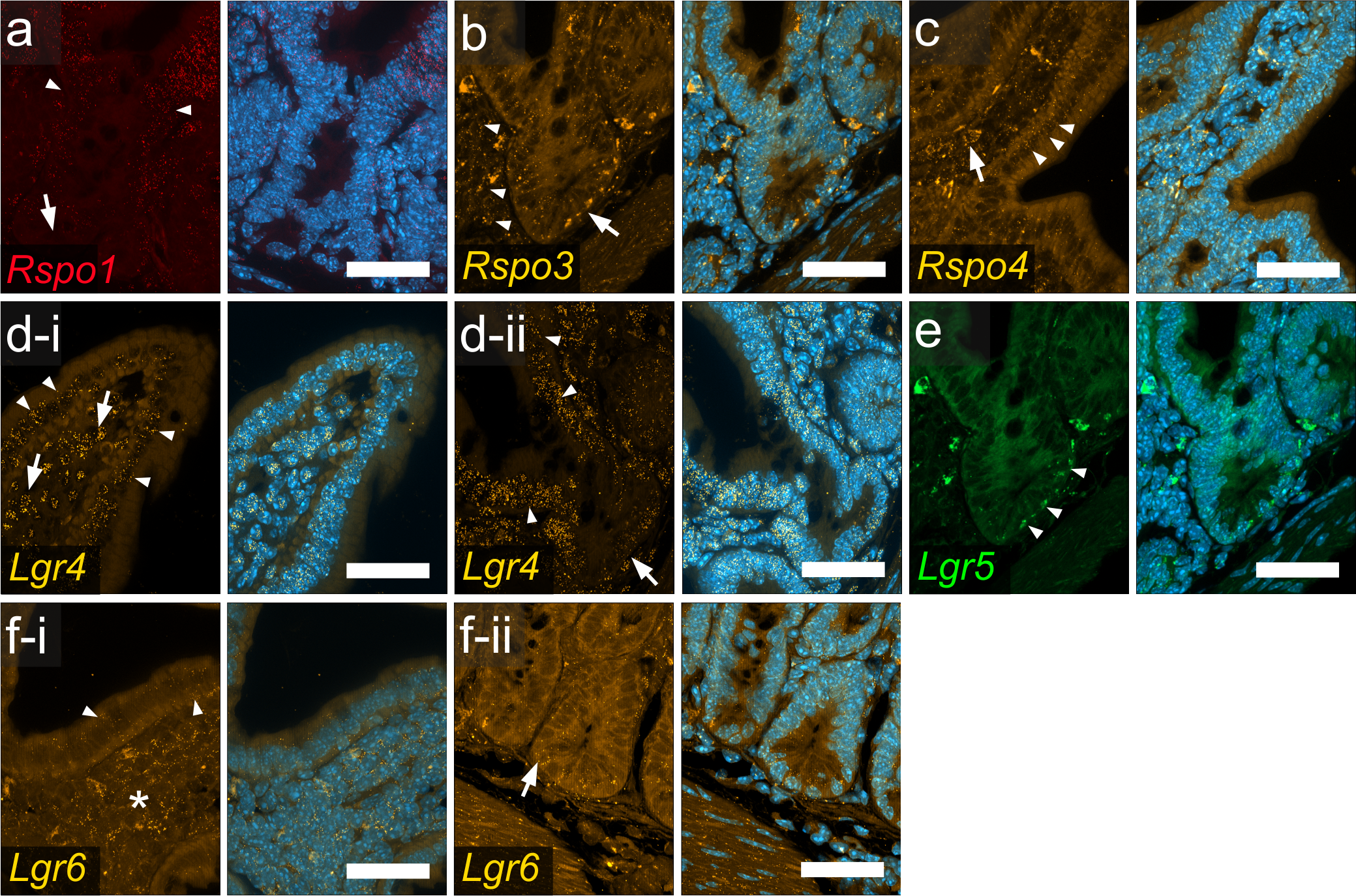

Supplement: Supplementary Figure 8 — R-spondin-ligands and Lgr-receptors are present in the murine small intestine. In situ hybridization experiments showed Rspo1- (A) , Rspo3- (B) , Rspo4- (C) , Lgr4- (D) , Lgr5- (E) , and Lgr6 (F) -mRNA detection (colors as indicated) and the nuclear marker DAPI (blue) within different compartments of the murine small intestine. Arrows, arrowheads, and asterisk indicate respectively for the corresponding transcript expressing or non-expressing cells. Scale bars: 40 µm. [file Image_8.tif]

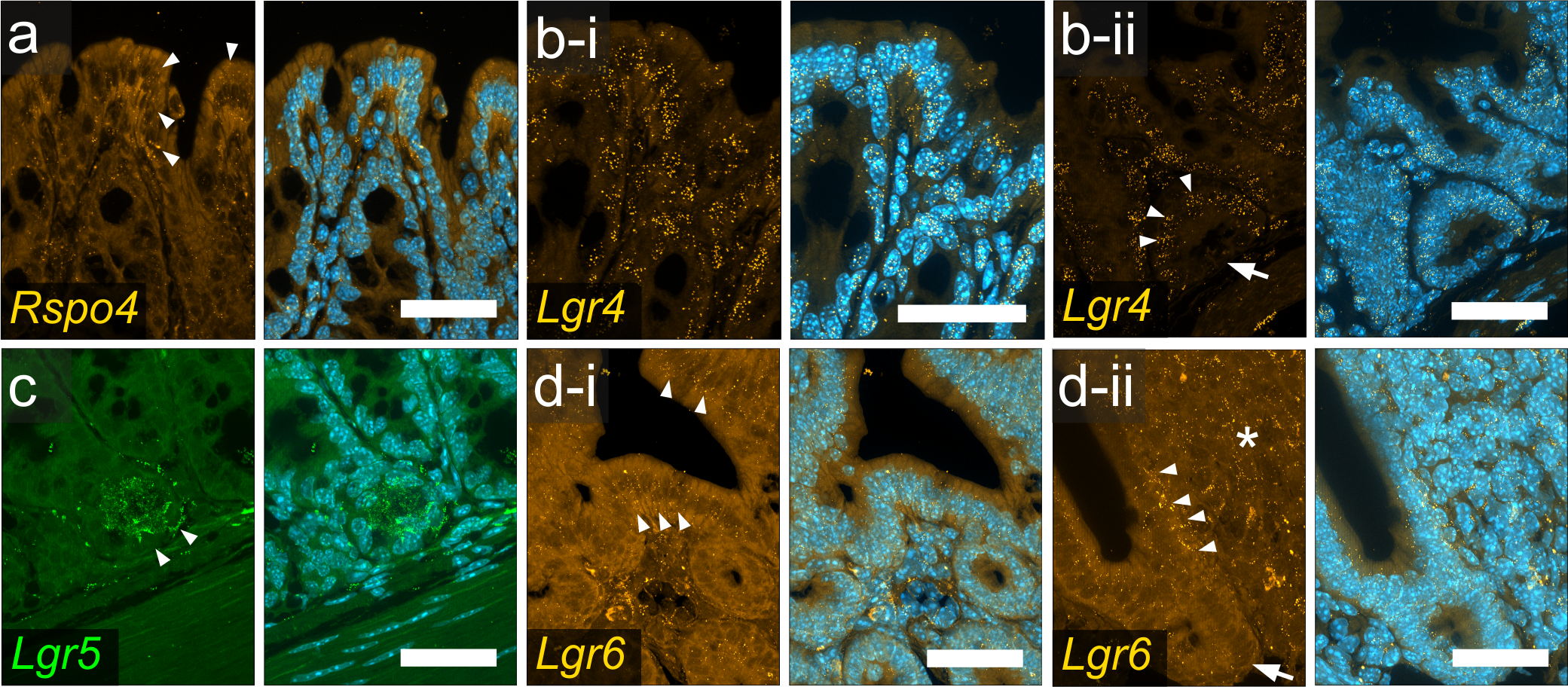

Supplement: Supplementary Figure 9 — R-spondin-ligands and Lgr-receptors are present in the murine large intestine. Representative images depict the expression of Rspo4- (A) , Lgr4- (B) , Lgr5- (C) , and Lgr6 (D) -mRNAs and the nuclear marker DAPI (blue) within the gastrointestinal wall of the large intestine. Arrows, arrowheads, and asterisk indicate respectively for the corresponding transcript expressing or non-expressing cells. Scale bars: 40 µm. [file Image_9.tif]
