# Supplementary material for: Spatial gene expression profile of Wnt-signaling components in the murine enteric nervous system
Source: Front Immunol. 2024 Jan 18;15:1302488. doi: 10.3389/fimmu.2024.1302488 (PMC10846065; doi:10.3389/fimmu.2024.1302488)
Supplement: Supplementary file 10 [file Table_1.docx]

**Supplementary Table 1:** Localization of mRNA targets in neuronal cell bodies versus ganglionic neuropil^#^ (Y = yes, N = no expression, nA = not assesable*).

| **mRNA targets** | **small intestine**   \| **SubG**  **neuron/neuropil** \| **MyG**  **neuron/neuropil** \| \| --- \| --- \| | **large intestine**   \| **Sub**  **neuron/neuropil** \| **MyG**  **neuron/neuropil** \| \| --- \| --- \| |
| --- | --- | --- | --- | --- | --- | --- |
| *Dkk1* | \| Y/Y \| Y/Y \| \| --- \| --- \| | \| Y/nA \| Y/Y \| \| --- \| --- \| |
| *Dkk2* | \| Y/nA \| Y/Y \| \| --- \| --- \| | \| Y/Y \| Y/Y \| \| --- \| --- \| |
| *Dkk3* | \| Y/nA \| Y/Y \| \| --- \| --- \| | \| Y/nA \| Y/Y \| \| --- \| --- \| |
| *Dkk4* | \| Y/nA \| Y/Y \| \| --- \| --- \| | \| Y/N \| Y/Y \| \| --- \| --- \| |
| *Fzd1* | \| Y/Y \| Y/Y \| neuron \| glia \| \| --- \| --- \| --- \| --- \| | \| Y/nA \| Y/Y \| neuron \| glia \| \| --- \| --- \| --- \| --- \| |
| *Fzd2* | \| Y/nA \| Y/Y \| \| --- \| --- \| | \| Y/Y \| Y/N \| \| --- \| --- \| |
| *Fzd3* | \| Y/nA \| Y/Y \| \| --- \| --- \| | \| Y/Y \| Y/N \| \| --- \| --- \| |
| *Fzd4* | \| Y/Y \| Y/Y \| \| --- \| --- \| | \| Y/Y \| Y/N \| \| --- \| --- \| |
| *Fzd5* | \| Y/Y \| Y/Y \| \| --- \| --- \| | \| Y/Y \| Y/Y \| \| --- \| --- \| |
| *Fzd6* | \| Y/nA \| Y/Y \| \| --- \| --- \| | \| Y/nA \| Y/Y \| \| --- \| --- \| |
| *Fzd7* | \| Y/nA \| Y/Y \| \| --- \| --- \| | \| Y/Y \| Y/N \| \| --- \| --- \| |
| *Fzd8* | \| Y/Y \| Y/N \| \| --- \| --- \| | \| Y/nA \| Y/Y \| \| --- \| --- \| |
| *Fzd9* | \| Y/Y \| Y/N \| \| --- \| --- \| | \| Y/nA \| Y/Y \| \| --- \| --- \| |
| *Fzd10* | \| Y/Y \| Y/N \| \| --- \| --- \| | \| N/N \| Y/Y \| \| --- \| --- \| |
| *Kremen1* | \| Y/Y \| Y/Y \| \| --- \| --- \| | \| Y/Y \| Y/Y \| \| --- \| --- \| |
| *Kremen2* | \| Y/nA \| Y/Y \| \| --- \| --- \| | \| Y/Y \| Y/Y \| \| --- \| --- \| |
| *Lgr4* | \| Y/Y \| Y/Y \| \| --- \| --- \| | \| Y/nA \| Y/Y \| \| --- \| --- \| |
| *Lgr5* | \| Y/Y \| Y/Y \| \| --- \| --- \| | \| Y/Y \| Y/Y \| \| --- \| --- \| |
| *Lgr6* | \| Y/Y \| Y/Y \| \| --- \| --- \| | \| Y/nA \| Y/Y \| \| --- \| --- \| |
| *Lrp5* | \| Y/Y \| Y/Y \| \| --- \| --- \| | \| Y/Y \| Y/Y \| \| --- \| --- \| |
| *Lrp6* | \| Y/Y \| Y/Y \| \| --- \| --- \| | \| Y/Y \| Y/Y \| \| --- \| --- \| |
| *Rspo1* | \| Y/Y \| Y/Y \| \| --- \| --- \| | \| Y/nA \| Y/N \| \| --- \| --- \| |
| *Rspo2* | \| Y/Y \| Y/Y \| \| --- \| --- \| | \| Y/Y \| Y/N \| \| --- \| --- \| |
| *Rspo3* | \| Y/Y \| Y/Y \| \| --- \| --- \| | \| Y/nA \| Y/Y \| \| --- \| --- \| |
| *Rspo4* | \| Y/Y \| Y/Y \| \| --- \| --- \| | \| Y/nA \| Y/Y \| \| --- \| --- \| |
| *Wls* | \| Y/nA \| Y/Y \| \| --- \| --- \| | \| Y/nA \| N/Y \| \| --- \| --- \| |
| *Wnt1* | \| Y/N \| Y/Y \| \| --- \| --- \| | \| Y/nA \| Y/Y \| \| --- \| --- \| |
| *Wnt2* | \| Y/nA \| Y/Y \| \| --- \| --- \| | \| Y/nA \| Y/Y \| \| --- \| --- \| |
| *Wnt2b* | \| Y/nA \| Y/Y \| \| --- \| --- \| | \| Y/nA \| Y/Y \| \| --- \| --- \| |
| *Wnt3* | \| Y/nA \| Y/Y \| \| --- \| --- \| | \| Y/nA \| Y/Y \| \| --- \| --- \| |
| *Wnt3a* | \| Y/Y \| Y/Y \| \| --- \| --- \| | \| Y/nA \| Y/Y \| \| --- \| --- \| |
| *Wnt4* | \| Y/Y \| Y/Y \| \| --- \| --- \| | \| Y/N \| Y/N \| \| --- \| --- \| |
| *Wnt5a* | \| Y/Y \| Y/Y \| \| --- \| --- \| | \| Y/nA \| Y/Y \| \| --- \| --- \| |
| *Wnt5b* | \| Y/nA \| Y/Y \| \| --- \| --- \| | \| Y/nA \| Y/Y \| \| --- \| --- \| |
| *Wnt6* | \| Y/Y \| Y/Y \| \| --- \| --- \| | \| Y/nA \| Y/Y \| \| --- \| --- \| |
| *Wnt7a* | \| N/N \| N/N \| \| --- \| --- \| | \| N/N \| N/N \| \| --- \| --- \| |
| *Wnt7b* | \| Y/Y \| Y/Y \| \| --- \| --- \| | \| Y/nA \| Y/Y \| \| --- \| --- \| |
| *Wnt8a* | \| Y/Y \| Y/Y \| \| --- \| --- \| | \| Y/nA \| Y/Y \| \| --- \| --- \| |
| *Wnt8b* | \| Y/Y \| Y/Y \| \| --- \| --- \| | \| Y/nA \| Y/Y \| \| --- \| --- \| |
| *Wnt9a* | \| Y/Y \| Y/Y \| \| --- \| --- \| | \| Y/nA \| Y/N \| \| --- \| --- \| |
| *Wnt9b* | \| N/N \| N/N \| \| --- \| --- \| | \| Y/nA \| Y/N \| \| --- \| --- \| |
| *Wnt10a* | \| Y/nA \| Y/Y \| \| --- \| --- \| | \| Y/nA \| Y/Y \| \| --- \| --- \| |
| *Wnt10b* | \| N/N \| N/N \| \| --- \| --- \| | \| N/N \| N/N \| \| --- \| --- \| |
| *Wnt11* | \| Y/Y \| Y/Y \| \| --- \| --- \| | \| N/N \| N/N \| \| --- \| --- \| |
| *Wnt16* | \| Y/Y \| Y/Y \| \| --- \| --- \| | \| Y/nA \| Y/Y \| \| --- \| --- \| |

^#^ Note: Specific markers for non-neural intraganglionic cells (e.g., macrophages) were not applied.

* No submucosal ganglia detectable on the respective tissue section.

**Supplementary Table 2:** Data-mining of Wnt related mRNA expression in scRNA-seq data published by Zeisel et al. (2018). Data available on http://loom.linnarssonlab.org/.

| **mRNA targets** | \| **neuronal cluster** \| **glial cluster** \| \| --- \| --- \| |
| --- | --- | --- | --- |
| *Dkk1* | \| ENT3, 8, 9 \| ENTG3, 5 \| \| --- \| --- \| |
| *Dkk2* | \| ENT1, 3, 7, 8 \| ENTG1, 3, 5 \| \| --- \| --- \| |
| *Dkk3* | \| ENT1-9 \| ENMFB, ENTG1-7 \| \| --- \| --- \| |
| *Dkk4* | \| not detectable \| not detectable \| \| --- \| --- \| |
| *Fzd1* | \| ENT1-9 \| ENMFB, ENTG1-7 \| \| --- \| --- \| |
| *Fzd2* | \| ENT1-6, 8-9 \| ENMFB, ENTG1-7 \| \| --- \| --- \| |
| *Fzd3* | \| ENT1-9 \| ENTG1-7 \| \| --- \| --- \| |
| *Fzd4* | \| ENT1-6, 9 \| ENMFB, ENTG1-7 \| \| --- \| --- \| |
| *Fzd5* | \| ENT2-6, 8-9 \| ENMFB, ENTG1-7 \| \| --- \| --- \| |
| *Fzd6* | \| ENT2, 3-5, 8 \| ENMFB, ENTG2-7 \| \| --- \| --- \| |
| *Fzd7* | \| ENT3-5, 9 \| ENMFB, ENTG1-7 \| \| --- \| --- \| |
| *Fzd8* | \| ENT2-9 \| ENMFB, ENTG1-7 \| \| --- \| --- \| |
| *Fzd9* | \| ENT5 \| ENTG3 \| \| --- \| --- \| |
| *Fzd10* | \| ENT3 \| ENTG1-5, 7 \| \| --- \| --- \| |
| *Kremen1* | \| ENT1-9 \| ENMFB, ENTG1-7 \| \| --- \| --- \| |
| *Kremen2* | \| ENT2, 9 \| ENTG2-7 \| \| --- \| --- \| |
| *Lgr4* | \| ENT1-9 \| ENMFB, ENTG1-7 \| \| --- \| --- \| |
| *Lgr5* | \| ENT1-6, 9 \| ENTG1, 2, 4, 6 \| \| --- \| --- \| |
| *Lgr6* | \| ENT8, 9 \| ENTG1-7 \| \| --- \| --- \| |
| *Lrp5* | \| ENT2-9 \| ENMFB, ENTG1-7 \| \| --- \| --- \| |
| *Lrp6* | \| ENT1-9 \| ENMFB, ENTG1-7 \| \| --- \| --- \| |
| *Rspo1* | \| ENT4, 6-9 \| ENMFB, ENTG2, 4, 5 \| \| --- \| --- \| |
| *Rspo2* | \| ENT2-9 \| ENTG1-2, 4-7 \| \| --- \| --- \| |
| *Rspo3* | \| ENT2-9 \| ENMFB, ENTG2-4, 6, 7 \| \| --- \| --- \| |
| *Rspo4* | \| ENT5, 8, 9 \| not detectable \| \| --- \| --- \| |
| *Wls* | \| ENT1-9 \| ENMFB, ENTG1-7 \| \| --- \| --- \| |
| *Wnt1* | \| ENT3, 8, 9 \| ENTG1, 2, 4-7 \| \| --- \| --- \| |
| *Wnt2* | \| ENT9 \| not detectable \| \| --- \| --- \| |
| *Wnt2b* | \| ENT6 \| not detectable \| \| --- \| --- \| |
| *Wnt3* | \| ENT1-3, 8 \| not detectable \| \| --- \| --- \| |
| *Wnt3a* | \| not detectable \| not detectable \| \| --- \| --- \| |
| *Wnt4* | \| ENT1-9 \| ENMFB, ENTG1-7 \| \| --- \| --- \| |
| *Wnt5a* | \| ENT1-9 \| ENMFB, ENTG1-7 \| \| --- \| --- \| |
| *Wnt5b* | \| not detectable \| ENMFB, ENTG1-4 \| \| --- \| --- \| |
| *Wnt6* | \| ENT2-9 \| ENMFB, ENTG1-7 \| \| --- \| --- \| |
| *Wnt7a* | \| not detectable \| not detectable \| \| --- \| --- \| |
| *Wnt7b* | \| not detectable \| ENTG4, 7 \| \| --- \| --- \| |
| *Wnt8a* | \| not detectable \| not detectable \| \| --- \| --- \| |
| *Wnt8b* | \| not detectable \| not detectable \| \| --- \| --- \| |
| *Wnt9a* | \| ENT1-9 \| ENMFB, ENTG1-7 \| \| --- \| --- \| |
| *Wnt9b* | \| not detectable \| not detectable \| \| --- \| --- \| |
| *Wnt10a* | \| not detectable \| ENTG2, 4, 6, 7 \| \| --- \| --- \| |
| *Wnt10b* | \| not detectable \| not detectable \| \| --- \| --- \| |
| *Wnt11* | \| ENT1-5, 7, 9 \| ENTG1-7 \| \| --- \| --- \| |
| *Wnt16* | \| ENT6 \| not detectable \| \| --- \| --- \| |

**Supplementary Table 3:** Data-mining of Wnt related mRNA expression in enteric glial cells using a RiboTag-based bulk RNA-seq published by Leven and Schneider et al. 2023. Samples were collected 3h, 24h, and 72h after intestinal manipulation to monitor the progress of enteric gliosis in a post-operative ileus model (early/immediate phase, inflammatory/manifestation phase, and recovery/resolution phase, respectively).

|  |  | **3h** | | **24 h** | | **72 h** | |
| --- | --- | --- | --- | --- | --- | --- | --- |
| **Gene symbol** | **Transcript ID** | **P-value ≤ 0.05 vs. naive** | **Fold change vs. naive** | **P-value ≤ 0.05 vs. naive** | **Fold change vs. naive** | **P-value ≤ 0.05 vs. naive** | **Fold change vs. naive** |
| *Dkk2* | Dkk2-201 | n | -1,77E+16 | y | -2,27E+15 | y | -4,04E+15 |
| *Dkk3* | Dkk3-201 | n | 1,36E+16 | y | -7,00E+15 | n.d. | n.d. |
| *Fzd1* | Fzd1-201 | n | -1,18E+15 | n.d. | n.d. | n.d. | n.d. |
| *Fzd2* | Fzd2-201 | y | -2,64E+16 | n | -2,00E+16 | y | -3,78E+15 |
| *Fzd3* | Fzd3-202 | n | 1,80E+14 | n | -1,92E+16 | n.d. | n.d. |
| *Fzd4* | Fzd4-201 | n | 1,31E+16 | n.d. | n.d. | n.d. | n.d. |
| *Fzd5* | Fzd5-201 | n | 1,77E+15 | n.d. | n.d. | n | -2,24E+16 |
| *Fzd6* | Fzd6-201 | n | -1,47E+15 | n.d. | n.d. | n.d. | n.d. |
| *Fzd6* | Fzd6-202 | n | 7,90E+15 | n.d. | n.d. | n.d. | n.d. |
| *Fzd7* | Fzd7-201 | n | -1,04E+16 | y | -4,07E+15 | n.d. | n.d. |
| *Fzd8* | Fzd8-201 | n | -1,03E+15 | n.d. | n.d. | n.d. | n.d. |
| *Kremen1* | Kremen1-202 | n | 1,05E+16 | n.d. | n.d. | n.d. | n.d. |
| *Kremen1* | Kremen1-201 | y | 2,70E+15 | n.d. | n.d. | n.d. | n.d. |
| *Lgr4* | Lgr4-202 | n | 1,66E+15 | n | -3,12E+16 | n.d. | n.d. |
| *Lgr4* | Lgr4-201 | n | 1,66E+15 | n | -3,12E+15 | n.d. | n.d. |
| *Lrp5* | Lrp5-201 | n | -1,05E+16 | n.d. | n.d. | n.d. | n.d. |
| *Lrp5* | Lrp5-203 | n | -2,97E+16 | n.d. | n.d. | n.d. | n.d. |
| *Lrp6* | Lrp6-204 | n | -1,08E+16 | n.d. | n.d. | n.d. | n.d. |
| *Lrp6* | Lrp6-205 | n | 1,65E+16 | n | -1,62E+16 | n.d. | n.d. |
| *Lrp6* | Lrp6-201 | n | 1,07E+16 | n.d. | n.d. | n.d. | n.d. |
| *Rspo1* | Rspo1-201 | n | -2,93E+16 | n.d. | n.d. | n.d. | n.d. |
| *Rspo2* | Rspo2-203 | n | 1,23E+16 | n.d. | n.d. | n.d. | n.d. |
| *Rspo3* | Rspo3-201 | y | 2,68E+16 | n.d. | n.d. | n.d. | n.d. |
| *Rspo3* | Rspo3-202 | n | -1,04E+16 | n | 1,21E+16 | n.d. | n.d. |
| *Sfrp1* | Sfrp1-201 | n | 1,50E+16 | y | -3,47E+16 | y | -1,65E+16 |
| *Sfrp5* | Sfrp5-201 | n | 1,38E+16 | n.d. | n.d. | n.d. | n.d. |
| *Wnt4* | Wnt4-201 | n | 1,17E+16 | n.d. | n.d. | n.d. | n.d. |
| *Wnt5a* | Wnt5a-201 | n | -1,37E+16 | n | -1,67E+16 | y | -3,07E+16 |
| *Wnt5b* | Wnt5b-202 | n | -1,24E+16 | n.d. | n.d. | n.d. | n.d. |
| *Wnt5b* | Wnt5b-201 | y | -8,70E+15 | n.d. | n.d. | n.d. | n.d. |
| *Wnt6* | Wnt6-201 | y | -3,78E+16 | n.d. | n.d. | n.d. | n.d. |

n.d. ≙ not detectable; y ≙ significant; n ≙ not significant
